# Multimarker Assessment of B-Cell and Plasma Cell Subsets and Their Prognostic Role in the Colorectal Cancer Microenvironment

**DOI:** 10.1158/1078-0432.CCR-24-4083

**Published:** 2025-04-08

**Authors:** Onni Sirkiä, Henna Karjalainen, Hanna Elomaa, Sara A. Väyrynen, Anne Tuomisto, Päivi Sirniö, Ville K. Äijälä, Vilja V. Tapiainen, Meeri Kastinen, Erkki-Ville Wirta, Olli Helminen, Sanna Meriläinen, Jukka Rintala, Juha Saarnio, Tero Rautio, Toni T. Seppälä, Markus J. Mäkinen, Jukka-Pekka Mecklin, Jan Böhm, Maarit Ahtiainen, Juha P. Väyrynen

**Affiliations:** 1 Department of Pathology, Hospital Nova of Central Finland, Well Being Services County of Central Finland, Jyväskylä, Finland.; 2 Department of Environmental and Biological Sciences, University of Eastern Finland, Kuopio, Finland.; 3 Translational Medicine Research Unit, University of Oulu, Medical Research Center Oulu, and Oulu University Hospital, Oulu, Finland.; 4 Department of Education and Research, Well Being Services County of Central Finland, Jyväskylä, Finland.; 5 Department of Internal Medicine, Oulu University Hospital, Oulu, Finland.; 6 Department of Gastroenterology and Alimentary Tract Surgery, Tampere University Hospital, Tampere, Finland.; 7 Faculty of Medicine and Health Technology, Tampere University and Tays Cancer Centre, Tampere University Hospital, Tampere, Finland.; 8 Department of Surgery, Oulu University Hospital, Oulu, Finland.; 9 Department of Gastrointestinal Surgery, Helsinki University Central Hospital, University of Helsinki, Helsinki, Finland.; 10 Applied Tumor Genomics, Research Program Unit, University of Helsinki, Helsinki, Finland.; 11 Faculty of Sport and Health Sciences, University of Jyväskylä, Jyväskylä, Finland.; 12 Central Finland Biobank, Hospital Nova of Central Finland, Well Being Services County of Central Finland, Jyväskylä, Finland.

## Abstract

**Purpose:**

Although the association between cytotoxic T lymphocytes and favorable prognosis in colorectal cancer is well established, the prognostic significance of B lymphocytes remains more ambiguous. This study aimed to assess the characteristics and significance of various B-cell and plasma cell subsets in colorectal tumors.

**Experimental Design:**

We designed a seven-plex IHC assay, combined with machine learning–based image analysis, to identify various B-cell and plasma cell populations and applied it to study a cohort of 912 colorectal tumors. We assessed the prognostic significance of B-cell and plasma cell densities using Kaplan–Meier estimators and Cox regression models. Additionally, we designed a more clinically applicable three-plex assay, which we used to study B-cell and plasma cell densities in a separate validation cohort of 737 patients.

**Results:**

High plasma cell density in the center of the tumor was associated with longer cancer-specific survival independent of disease stage, mismatch repair status, T-cell densities, and other covariates. In the study cohort, the multivariable HR for high (vs. low) plasma cell density was 0.48 (95% confidence interval, 0.32–0.72; *P*_trend_ = 0.0005), whereas the corresponding HR in the validation cohort was 0.37 (95% confidence interval, 0.21–0.65; *P*_trend_ = 0.0003). Of the specific subsets, IgG1^–^IgG2^–^ plasma cells showed the strongest association with improved survival. High B-cell densities were not independently associated with a better prognosis.

**Conclusions:**

Plasma cell densities in the center of the tumor represent a relevant tumor-immune biomarker in colorectal cancer, complementing T-cell density measurements.

**Translational Relevance**

This study highlights the significance of plasma cell densities as a tumor–immune biomarker in colorectal cancer. Using a custom seven-plex IHC assay combined with machine learning–based image analysis, we identified individual B-cell and plasma cell subsets and their spatial localization within the tumor microenvironment of 912 colorectal cancers. High plasma cell density in the tumor center emerged as a strong, independent predictor of improved cancer-specific survival, complementing established biomarkers such as T-cell densities and mismatch repair status. This result was validated in an independent cohort of 737 patients using a clinically applicable three-plex IHC assay. These findings support the integration of plasma cell density analysis into clinical practice, paving the way for enhanced risk stratification and therapeutic development in colorectal cancer.

## Introduction

Colorectal cancer is the third most commonly diagnosed and the second most deadly cancer worldwide with more than 1.9 million new diagnoses and more than 900,000 deaths in 2022 (1). The prognostic classification of colorectal cancer is mainly based on disease stage, but the vast heterogeneity of patient and disease characteristics may result in significantly different clinical outcomes within the same stage (2, 3). Several genetic factors, including microsatellite instability and both *RAS* and *BRAF* mutation status, also affect colorectal cancer prognosis, along with morphologic factors such as tumor differentiation and lymphovascular invasion (3–5). In addition, immune cell infiltrates of both the innate and adaptive immunity have been shown to harbor prognostic value in colorectal cancer (4–8). Furthermore, tertiary lymphoid structures (TLS) containing adaptive immune cells have also been associated with improved colorectal cancer prognosis (9, 10).

Cytotoxic T lymphocytes represent one of the most extensively studied subsets of tumor-infiltrating immune cells. High infiltration of cytotoxic T cells in colorectal tumors has been associated with better prognosis and diminished colorectal cancer recurrence (11, 12). These observations have given rise to the Immunoscore, a prognostic tool validated by a worldwide task force (13, 14), which is based on computer-assisted quantification of CD3^+^ and CD8^+^ T cells in the tumor center (CT) and the invasive margin (IM; ref. 13). In colorectal cancer, the Immunoscore and Immunoscore-like approaches seem to be valuable additional prognostic tools complementing the traditional tumor–node–metastasis classification (14–16).

The prognostic role of tumor-infiltrating B lymphocytes, in turn, has been much more contradictory, and these cells have been linked with both tumor-promoting and tumor-inhibiting effects. This variation has been attributed to the different populations of the B-lymphocyte lineage, potentially performing different functions in the humoral immune response and immunoregulation (17–19). Tumor-infiltrating B cells have been shown to frequently express a mature antigen-presenting phenotype, but the proportion of immunosuppressive regulatory B-cell subsets can often be higher in more advanced tumors (17, 18). The prognostic role of tumor-infiltrating plasma cell subsets, in turn, may depend on the isotype of the immunoglobulin they produce (18, 19). These factors could explain why the use of general B-cell or plasma cell markers, such as CD20 or CD138, has resulted in incoherent findings in different studies, with some supporting the independent prognostic role of B-lymphocyte lineage and others not (20–23).

The aim of this study was to investigate the prognostic importance of B-cell and plasma cell infiltrates in colorectal cancer. We utilized seven-plex IHC together with machine learning–based image analysis to identify various subsets of B cells and plasma cells within colorectal tumors of a large cohort (*n* = 912). Our primary aim was to evaluate whether either B-cell or plasma cell density scoring could be used as an independent prognostic factor in colorectal cancer. In addition, we wanted to assess the spatial localization of B cells and plasma cells in the tumors. Based on these analyses, plasma cell densities in the CT emerged as an independent prognostic parameter, and we validated their prognostic value in an independent cohort of 737 patients with colorectal cancer using a three-plex IHC assay that would be easier to apply to the clinical workflow than the more complex seven-plex IHC assay.

## Materials and Methods

### Patients

The main study cohort consisted of 1,343 patients who underwent colorectal cancer resection at the Central Finland Central Hospital from 2000 to 2015 and had adequate tumor samples available. The population in the Central Finland region averaged 270,000 during the study period (24). Relevant clinical and patient follow-up data were retrospectively gathered from the clinical records of the Central Finland Central Hospital. DNA mismatch repair (MMR) status and *BRAF*^V600E^ mutation status of all the tumors had been previously determined by IHC, whereas the T-cell density score (low, intermediate, or high) had been evaluated in advance using CD3 and CD8 IHC combined with digital image analysis and a method analogous to the Immunoscore. In brief, the densities of CD3^+^ and CD8^+^ T cells were determined by dividing cell counts by the analyzed tissue area in the CT and IM. These four density values were converted into percentiles, averaged, and categorized into T-cell density scores as low (0–25), intermediate (>25–70), or high (>70–100) based on the mean percentile (25). The densities of CD8^–^ T cells were approximated by subtracting CD8^+^ T-cell densities from CD3^+^ T-cell densities. This population approximately corresponds to T helper cells, considering that most T cells express either CD8 or CD4, and double-negative (CD8^–^CD4^–^) T cells are rare (26).

Histologic tumor parameters, including lymphovascular invasion and tumor differentiation, were reevaluated from hematoxylin and eosin–stained whole slides by the study pathologist (J.P. Väyrynen), who was blinded to the clinical data. The density of TLSs was calculated as the number of TLSs divided by the length of the invasive front, according to the established criteria (27). For this study, multiplex IHC (mIHC) was conducted, and patients with unrepresentative samples for either the CT or IM (*n* = 222) were excluded from the analyses. In addition, patients who had died within 30 days of the resection (*n* = 30) were also excluded from the study cohort, along with those who had received any preoperative oncological treatments (chemotherapy, radiotherapy, or chemoradiotherapy; *n* = 179), as these may have a major effect on the tumor characteristics and immune infiltrates (28, 29), resulting in a final study cohort of 912 patients. A flow diagram summarizing the patient inclusion criteria for the main study cohort is shown in Supplementary Fig. S1. The study was performed in accordance with the guidelines of the Declaration of Helsinki. The study was approved by the Regional Medical Research Ethics Committee of the Wellbeing Services County of Central Finland (Dnro 13U/2011, 1/2016, 8/2020, and 2/2023), the Finnish Medicines Agency (Fimea), and the Central Finland Biobank (BB23-0172). The need to obtain informed consent from the study patients was waived (Dnro FIMEA/2023/001573, 4/2023). The representativeness of the study participants is summarized in Supplementary Table S1.

### mIHC

We developed a mIHC assay to identify various B-cell and plasma cell subsets. CD20 and CD79A were used for detecting B cells (CD20^+^CD79A^+^) and plasma cells (CD20^–^CD79A^+^), as suggested in the literature (23, 30). In addition, we included IRF4 to assist in plasma cell detection and identify memory B cells (31, 32). Other markers included in the mIHC panel were HLA-DR in order to assess the antigen presentation of B cells; IgG1, IgG2, and IgG4 to determine the specific isotype of plasma cells; and cytokeratin (CK) to identify tumor cells (18, 19, 33). The final mIHC panel is shown in Supplementary Table S2, and the details of key resources are listed in Supplementary Table S3.

We utilized previously designed tissue microarray (TMA) blocks that contained four 1-mm diameter cores per tumor: two from the CT and two more from the IM. CT cores were selected to best represent the overall tumor morphology while avoiding necrotic areas. IM cores were chosen to span 500 µm into the tumor and 500 µm into the surrounding healthy tissue (25). The assay was performed using the Bond-III Automated IHC Immunostainer (Leica Biosystems; RRID: SCR_026521) using the BOND Polymer Refine Detection kit (DS9800, Leica Biosystems). A previously validated cyclic method utilizing 3-amino-9-ethylcarbazole (AEC; AEC^+^ high sensitivity substrate chromogen, K3469, Dako) as the chromogen was used (34). Before the first mIHC staining cycle, the 3.5-µm-thick TMA sections underwent a bake and dewax protocol presented in Supplementary Table S4. Additionally, heat-induced antigen retrieval (Supplementary Table S5) was performed before each staining cycle (Supplementary Table S2). mAbs used in the immunostainings were diluted with Leica BOND Primary Antibody Diluent (AR9352, Leica Biosystems). We optimized the dilutions based on standard IHC on a test TMA, including samples from tonsils, normal colorectal mucosa, and colorectal tumors. The correspondence of mIHC and standard IHC was assessed visually for all antibodies, with examples shown in Supplementary Fig. S2. The IgG4 stain was excluded from the final analysis because of the inadequate performance of the IgG4 antibody (RRID: AB_3676660) in the mIHC assay; thus, the final mIHC panel included seven markers.

After each immunostaining cycle, slides were manually covered with microscope cover glasses using VectaMount AQ (H-5501, Vector Laboratories) and scanned using NanoZoomer-XR (Hamamatsu Photonics; RRID: SCR_026520; resolution 0.45 µm/pixel). After the first (Supplementary Table S6) and final staining cycles, slides were manually destained using an ascending series of ethanol to obtain clean hematoxylin-stained images for image analysis. In all other cycles, destaining was performed as the initial steps of the subsequent staining cycle (steps 1–5 in Supplementary Tables S7–S8). The cover glasses were detached in a water bath overnight between the staining cycles.

### Image analysis

Digitized mIHC images were processed using QuPath software (version 0.3.0; RRID: SCR_018257; ref. 35). TMA cores were separated using the TMA dearrayer function and exported as individual images. The core images were assessed, and any cores that were folded, torn, necrotic, or contained no tumor tissue were removed. The AEC color channels of representative cores were then converted into pseudocolor images and merged into mIHC images by aligning hematoxylin images in Fiji software (RRID: SCR_002285; ref. 36). Examples of merged mIHC images are shown in Supplementary Fig. S2. All 25 TMA slides showed relatively uniform staining intensities (Supplementary Fig. S3), indicating successful immunostainings and comparable results across the TMAs.

**Figure 1.**
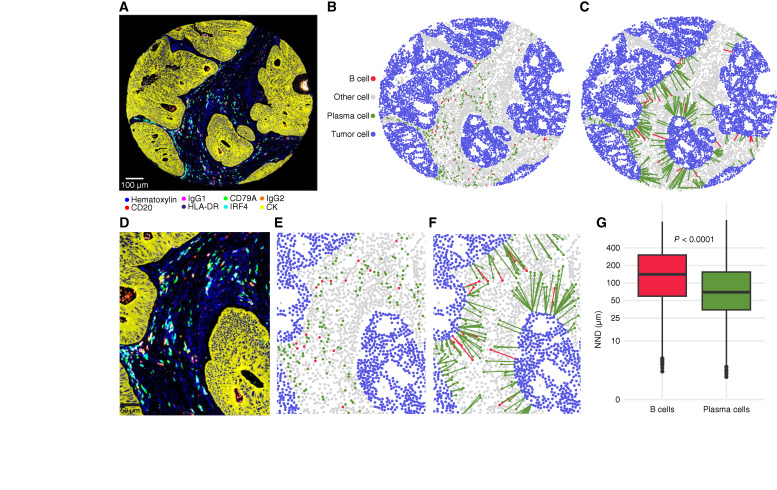
mIHC to identify various B-cell and plasma cell populations and their distribution in the colorectal cancer microenvironment. **A** and **D,** mIHC image of a representative example tumor. **B** and **E,** Image analysis result output showing the main cell types identified in the mIHC image. **C** and **F,** NND plot showing NNDs from individual B and plasma cells to the closest tumor cell in the example image. **G,** Box plot showing the distribution of NNDs from each B cell and plasma cell to the closest tumor cell across all tumors of the main study cohort (*n* = 912). The box plot is based on 501,460 plasma cells and 184,653 B cells. The *P* value was calculated using the Wilcoxon rank-sum test.

The mIHC images were analyzed with QuPath using a previously validated supervised machine learning approach (7). We trained the classifiers by manually annotating representative areas from 39 tumor samples with varying histology. Cell detection was carried out using the cell detection function, and both intensity and smoothed features were calculated for each cell. Using the object classifier function, we trained the software to identify B cells that coexpress CD20 and CD79A, plasma cells that coexpress CD79A and IRF4 without the presence of CD20, and tumor cells characterized by CK expression (30, 31, 33). Any cells falling outside these criteria were classified as “other.” The data provided us with the phenotypes (B/plasma/tumor/other cell) together with the marker intensities and coordinates for each individual cell. In addition, we utilized the pixel classifier function to train the software to segregate tumor epithelial regions from stromal regions based on CK expression and to exclude necrotic regions, white space, and folded tissue. The parameters used for image analysis in QuPath are given in Supplementary Table S9. Examples of cell identification and tissue categorization output are shown in Fig. 1 and Supplementary Fig. S4.

### Further immune cell phenotyping

The cell-level data obtained from image analysis were further processed with the R programming language (version 4.1.1, R Core Team; RRID: SCR_001905) and RStudio (version 1.4.1717, RStudio Team; RRID: SCR_000432) using the tidyverse package (version 1.3.1). Appropriate cutoff values for defining the positive and negative subsets were visually assessed from the images and marker intensity distribution plots. Nuclear intensity was used for IRF4 (31), and cytoplasmic intensities were used for IgG1, HLA-DR, and IgG2. Plasma cells were phenotyped into IgG1^+/–^, IgG2^+/–^, and IgG1^–^IgG2^–^ subsets to assess the prognostic role of both IgG1 and IgG2 isotypes and the remaining IgG1^–^IgG2^–^ plasma cell population (19, 30). B cells, in turn, were first phenotyped into IRF4^+/–^ and HLA-DR^+/–^ subsets and further into IRF4^+^HLA-DR^+^, IRF4^–^HLA-DR^+^, and IRF4^–^HLA-DR^–^ subsets corresponding to the memory B cells, activated antigen-presenting B cells, and naïve B cells, respectively (17, 18). In addition, the phenotyping resulted in a small portion of IgG1^+^IgG2^+^ plasma cells and IRF4^+^HLA-DR^–^ B cells (1.9% and 4.4%, respectively) that should not be possible based on the literature (17–19, 31). These seemed to represent technical artifacts (i.e., two cells overlapping each other in the IHC images) and were thus excluded from the later analyses. Immunophenotypes of various B-cell and plasma cell subsets with examples of individual stains and the mIHC overlay are shown in Supplementary Fig. S5, whereas a summary of marker combinations used for identifying various immune cell subsets is presented in Supplementary Table S10.

The densities (cells/mm^2^) of plasma cell and B-cell subsets were calculated separately for the tumor epithelial and stromal regions of each sample. Additionally, overall cell densities representing the entire sample (both tumor epithelial and stromal regions together) were calculated. All the cell densities were divided into four ordinal quartile categories (Q1–Q4), with Q1 representing the lowest density.

### Spatial analyses

Cell-level data were processed using the spatstat (version 2.2-0) R library to calculate the nearest neighbor distances (NND) from each B cell/plasma cell to the closest tumor cell. The intensities of various markers as a function of the NNDs in B cells/plasma cells in all images were plotted with ggplot (version 3.3.4) using generalized additive model smoothing [formula = y ∼ s(x)].

### Validation cohort

To further validate our finding of the independent prognostic value of plasma cell densities in the CT, we included an independent validation cohort that was prospectively collected at Oulu University Hospital between the years 2006 and 2020 (37). The analysis was based on TMAs that included four cores of 1.0 mm diameter: two from the CT and two more from the IM. In addition, 30 whole-slide sections were analyzed. Of the 1,014 patients with colorectal cancer, those who had unrepresentative samples for either the CT or IM (*n* = 70), received neoadjuvant treatment (*n* = 202), or died within 30 days of surgery (*n* = 5) were excluded, resulting in 737 patients in the final analyses. A flow diagram summarizing the patient inclusion criteria for the validation cohort is presented in Supplementary Fig. S1. The validation study was approved by the Regional Medical Research Ethics Committee of the Wellbeing Services County of North Ostrobothnia (25/2002, 42/2005, 122/2009, and 37/2020), Fimea (FIMEA/2022/001941), and Biobank Borealis (BB-2017_1012). Patients provided written informed consent for the study.

To facilitate potential clinical adoption of plasma cell densities as a prognostic marker, we developed a three-plex chromogenic assay (Supplementary Table S11) for their identification. The assay included CD20 and CD79A for detecting B cells and plasma cells and additionally CK to distinguish tumor epithelial and stromal regions. The images were analyzed using QuPath (version 0.5.1) with the same principles as in the main cohort, and the data were processed with R statistical programming to calculate cell densities per mm^2^ that were classified into ordinal quartile categories for survival analyses, following the example of the main cohort. In addition, CD138 IHC (Supplementary Table S12) was performed as a comparison with the developed three-plex assay and analyzed using similar principles as the three-plex assay.

### Statistical analyses

Statistical analyses were carried out using the R programming language and RStudio using corrplot (version 0.92), ggpubr (version 0.4.0), gmodels (version 2.18.1), survival (version 3.2–7), and survminer (version 0.4.9), in addition to the tidyverse and spatstat packages.

The associations of continuous overall plasma cell or B-cell density with patient characteristics (as well as NNDs with cell categories) were studied using either the Wilcoxon rank-sum test or the Kruskal–Wallis test. Spearman correlation coefficients were used to examine the correlations between different plasma cell and B-cell subset densities and also with the T-cell density data collected in the previous study (25).

In our primary aim, we utilized uni- and multivariable Cox proportional hazard regression models to determine HR point estimates and 95% confidence intervals (CI) for cancer-specific and overall survival (CSS and OS, respectively). CSS was the primary outcome and was defined as the time from surgery to colorectal cancer death (a death in which colorectal cancer is listed as the underlying cause of death in accordance with the International Classification of Diseases-10 coding principles). OS was the secondary outcome and was defined as the time from surgery to any death. The follow-up was limited to 10 years, considering that most colorectal cancer deaths occur within 10 years, and Schoenfeld residual plots supported the proportionality of hazards within this period. Multivariable models included the following predetermined indicator variables (with the reference category listed first): sex (male, female), age (<65, 65–75, >75), year of operation (2000–2005, 2006–2010, 2011–2015; validation cohort: 2006–2010, 2011–2015, 2016–2020), tumor location (proximal colon, distal colon, rectum), American Joint Committee on Cancer disease stage (I–II, III, IV), tumor grade (low-grade, high-grade), lymphovascular invasion (negative, positive), MMR status (proficient, deficient), *BRAF* status (wild-type, mutant), and T-cell density score (low, intermediate, high, missing). Kaplan–Meier estimators were used to visualize CSS between the ordinal quartile categories of plasma cells and B cells, and the statistical significance was tested using the log-rank test.

The threshold of statistical significance was set to 0.5% (*P* < 0.005) for all statistical analyses, as recommended by a panel of experts (38).

### Data availability

The data generated and/or analyzed during this study are not publicly available due to the confidentiality of patient data. The sharing of data will require approval from relevant ethics committees and/or biobanks. Further information, including the procedures to obtain and access data from Finnish Biobanks, is described at https://finbb.fi/en/fingenious-service. Requestors will need to register with Finnish Biobanks to begin the data request process, and additional questions can be directed to the corresponding authors.

## Results

### Image analysis identifies individual B cells and plasma cells and their spatial localization in the tumor microenvironment

By using a supervised machine learning–based approach on the seven-plex mIHC images (Fig. 1), we analyzed 3,168 TMA cores (mean 3.5 per case, SD 0.69) from 912 patients with colorectal cancer with a median age of 72 years at the time of diagnosis. Our cohort had a male-to-female ratio of 1:1, and the patients represented all American Joint Committee on Cancer stages (16.8%, 36.9%, 33.6%, and 12.7% of stages I, II, III, and IV, respectively). The main clinicopathologic characteristics of the patients are presented in Table 1. Our analysis confirmed that patients who received preoperative treatment had lower B-cell densities and those who received chemoradiotherapy had higher plasma cell densities (Supplementary Fig. S6), thereby justifying their removal from the main study cohort. The total number of identified cells was 18,656,391, including 501,460 (2.7%) plasma cells, 184,653 (0.99%) B cells, and 9,784,384 (52.4%) tumor cells. IgG1^+^ and IgG2^+^ subsets accounted for 25.6% and 11.3%, respectively, of all plasma cells, whereas IRF4^+^HLA-DR^+^ memory B cells and antigen-presenting IRF4^–^HLA-DR^+^ B cells accounted for 21.4% and 53.1%, respectively, of the total B-cell population. Densities of various plasma cell and B-cell subsets are shown in Supplementary Fig. S5. Core-to-core correlations for plasma cell and B-cell subsets were moderate to good (Supplementary Figs. S7 and S8). Densities of plasma cell subsets generally showed stronger correlations with other plasma cells, whereas B-cell subsets were more strongly correlated with other B cells than with T cells or other cell types (Supplementary Fig. S9).

**Table 1.** Clinicopathologic characteristics of colorectal cancer cases according to plasma cell and B-cell densities in the CT and IM.

		Overall plasma cell density (cells/mm^2^)Median (25th–75th percentiles)				Overall B-cell density (cells/mm^2^)Median (25th–75th percentiles)			
Characteristic	Total *n*	CT	*P*	IM	*P*	CT	*P*	IM	*P*
All cases	912	57 (19–160)		101 (32–252)		6.3 (1.7–25)		22 (5.7–83)	
Sex			0.72		0.48		0.51		0.40
Female	456	57 (19–170)		97 (32–242)		6.9 (2.0–25)		21 (5.5–76)	
Male	456	57 (20–151)		111 (34–262)		6.1 (1.5–25)		23 (6.0–91)	
Age (years)			0.42		0.34		0.33		0.48
<65	245	54 (19–151)		103 (36–220)		7.3 (1.8–25)		23 (5.9–113)	
65–75	328	56 (17–144)		116 (35–282)		5.4 (1.6–24)		22 (5.6–86)	
>75	339	60 (22–192)		88 (30–234)		6.9 (2.0–25)		21 (5.7–74)	
Year of operation			0.97		0.77		0.35		0.11
2000–2005	271	57 (20–162)		104 (31–278)		6.3 (1.7–24)		22 (5.6–77)	
2006–2010	295	56 (21–152)		101 (32–234)		5.5 (1.7–19)		19 (4.4–65)	
2011–2015	346	57 (18–167)		100 (34–260)		7.3 (1.8–28)		24 (6.7–97)	
Tumor location			0.027		0.0008		0.95		0.0016
Proximal colon	448	65 (22–194)		126 (39–311)		6.2 (1.8–26)		29 (7.2–97)	
Distal colon	336	47 (16–137)		86 (29–219)		6.7 (1.7–24)		17 (5.3–67)	
Rectum	128	63 (23–137)		82 (29–188)		7.0 (1.5–24)		18 (4.2–75)	
AJCC stage			0.0005		0.0032		0.0086		0.0048
I	153	87 (28–266)		146 (42–316)		12 (3.3–34)		28 (10–111)	
II	337	59 (20–162)		123 (36–294)		5.9 (2.1–19)		25 (5.8–80)	
III	306	50 (17–136)		85 (31–221)		5.4 (1.2–25)		21 (5.0–87)	
IV	116	47 (14–118)		68 (27–173)		7.5 (1.6–25)		13 (3.2–37)	
Tumor grade			0.61		0.031		0.58		0.0002
Low-grade	760	58 (20–154)		99 (30–244)		6.3 (1.9–23)		20 (5.2–74)	
High-grade	152	54 (13–190)		131 (45–316)		8.0 (1.1–34)		36 (12–130)	
Lymphovascular invasion			0.24		0.0037		0.86		0.68
No	717	59 (20–164)		110 (36–280)		6.6 (1.8–25)		22 (5.9–83)	
Yes	195	52 (16–146)		72 (25–183)		6.0 (1.4–25)		21 (5.0–79)	
MMR status			0.96		<0.0001		0.73		<0.0001
MMR proficient	771	58 (19–154)		92 (30–223)		6.3 (1.8–24)		19 (4.6–74)	
MMR deficient	141	54 (19–183)		194 (55–393)		6.9 (1.7–28)		45 (15–132)	
*BRAF* status			0.69		0.0011		0.61		0.0051
Wild-type	759	57 (20–155)		97 (30–239)		6.5 (1.8–25)		20 (4.9–81)	
V600E mutant	153	56 (16–171)		154 (55–330)		5.8 (1.6–25)		32 (12–95)	

Abbreviation: AJCC, American Joint Committee on Cancer.

*P* values were calculated using either the Wilcoxon rank-sum test (two groups) or the Kruskal–Wallis test (three/four groups).

Spatially, plasma cells were located, on average, 51% closer to tumor cells than the B cells (Fig. 1). Of the plasma cell populations, IgG1^–^ and IgG2^–^ cells were closer to tumor cells than IgG1^+^ and IgG2^+^ cells, whereas of the B-cell populations, IRF4^+^ and HLA-DR^+^ cells were closer than IRF4^–^ and HLA-DR^–^ cells (Supplementary Fig. S10).

The associations of plasma cell and B-cell densities with patient and tumor characteristics are displayed in Table 1. Higher plasma cell densities in both the CT and IM were associated with the disease stage. In addition, higher densities of both plasma cells and B cells in the IM were associated with proximal tumor location, mutant *BRAF* status, and MMR deficiency. Lymphovascular invasion showed an association with decreased plasma cell density (*P* = 0.0037) but not with B-cell density (*P* = 0.68) in the IM, whereas no statistically significant associations were observed in the CT. High tumor grade was associated with higher B-cell density (*P* = 0.0002) but not significantly with plasma cell density (*P* = 0.031) in the IM.

### High plasma cell densities in the center of the tumor represent an independent favorable prognostic parameter

**Figure 2.**
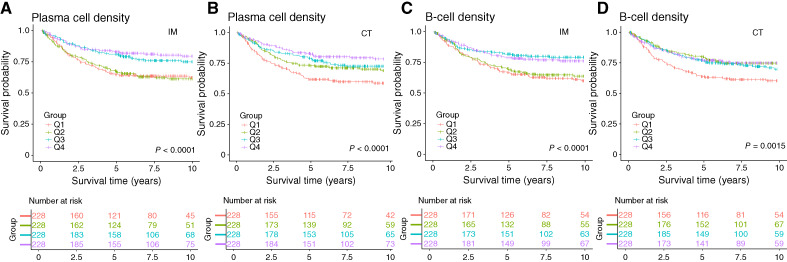
Kaplan–Meier estimates of CSS in the main cohort. The curves visualize CSS according to the ordinal quartile categories (Q1–Q4, from low to high) of overall plasma cell densities in the IM (**A**) and CT (**B**) and overall B-cell densities in the IM (**C**) and CT (**D**). *P* values were calculated using the log-rank test.

In our main analysis, we evaluated the prognostic effect of various plasma cell and B-cell populations. The number of deaths was 455, including 248 cancer deaths, and the median follow-up time for censored cases was 10 years (IQR, 7.1–10). Kaplan–Meier estimators (Fig. 2) and univariable Cox regression models (Table 2) indicated that high densities of plasma cells and B cells in both the CT and IM were associated with longer CSS. In multivariable analyses, high plasma cell density in the CT was associated with favorable survival independent of covariates, including but not limited to disease stage, T-cell density score, and MMR status, whereas B cells had no independent prognostic value either in the CT or IM (Table 2; Supplementary Tables S13 and S14). The multivariable HR for high (vs. low) plasma cell density in the CT was 0.48 (95% CI, 0.32–0.72; *P*_trend_ = 0.0005). Given that most B cells and plasma cells reside in the tumor stroma, we conducted additional analyses on stromal immune cell densities (Supplementary Fig. S11; Supplementary Table S15), and the results were quite similar to the main analyses that did not differentiate between tumor epithelial and stromal regions. In a sensitivity analysis, we assessed the prognostic value of B-cell and plasma cell densities as binary variables (Supplementary Table S16; Supplementary Fig. S12). Using above/below median classification, B-cell and plasma cell densities in the IM were significantly associated with longer CSS in addition to plasma cells in the CT. The prognostic significance of plasma cells did not differ significantly by tumor–node–metastasis stage (Supplementary Table S17), and plasma cell densities improved patient stratification when added to T, N, and M stages (Supplementary Fig. S13).

**Table 2.** Uni- and multivariable Cox regression models for CSS and OS according to overall plasma cell and B-cell densities in the CT and IM in the study cohort.

		CSS			OS		
	No. of cases	No. of events	Univariable HR (95% CI)	Multivariable HR (95% CI)	No. of events	Univariable HR (95% CI)	Multivariable HR (95% CI)
Plasma cells							
CT							
Q1	228	85	1 (referent)	1 (referent)	135	1 (referent)	1 (referent)
Q2	228	64	0.68 (0.49–0.94)	0.66 (0.47–0.93)	110	0.72 (0.56–0.93)	0.69 (0.53–0.90)
Q3	228	56	0.58 (0.42–0.82)	0.64 (0.45–0.93)	108	0.68 (0.53–0.88)	0.73 (0.56–0.96)
Q4	228	43	0.44 (0.31–0.64)	0.48 (0.32–0.72)	102	0.64 (0.49–0.82)	0.65 (0.49–0.87)
*P*_trend_			<0.0001	0.0005		0.0006	0.0093
IM							
Q1	228	78	1 (referent)	1 (referent)	126	1 (referent)	1 (referent)
Q2	228	78	1.01 (0.74–1.38)	1.04 (0.76–1.44)	128	1.02 (0.80–1.31)	1.07 (0.83–1.37)
Q3	228	50	0.58 (0.41–0.83)	0.66 (0.46–0.97)	105	0.73 (0.56–0.95)	0.81 (0.62–1.06)
Q4	228	42	0.48 (0.33–0.69)	0.67 (0.45–1.00)	96	0.65 (0.50–0.85)	0.78 (0.59–1.03)
*P*_trend_			<0.0001	0.010		0.0002	0.026
B cells							
CT							
Q1	228	83	1 (referent)	1 (referent)	133	1 (referent)	1 (referent)
Q2	228	52	0.56 (0.40–0.80)	0.72 (0.50–1.03)	103	0.68 (0.53–0.88)	0.77 (0.59–1.00)
Q3	228	61	0.65 (0.47–0.90)	0.75 (0.53–1.08)	114	0.75 (0.58–0.96)	0.84 (0.64–1.09)
Q4	228	52	0.58 (0.41–0.82)	0.71 (0.49–1.04)	105	0.73 (0.56–0.94)	0.84 (0.64–1.10)
*P*_trend_			0.0046	0.089		0.031	0.28
IM							
Q1	228	82	1 (referent)	1 (referent)	127	1 (referent)	1 (referent)
Q2	228	74	0.90 (0.66–1.23)	0.96 (0.69–1.32)	121	0.95 (0.74–1.22)	0.92 (0.72–1.19)
Q3	228	43	0.50 (0.34–0.72)	0.65 (0.44–0.96)	106	0.78 (0.60–1.00)	0.85 (0.64–1.12)
Q4	228	49	0.56 (0.39–0.80)	0.73 (0.49–1.08)	101	0.73 (0.56–0.95)	0.88 (0.66–1.17)
*P*_trend_			<0.0001	0.039		0.0063	0.31

Multivariable Cox proportional hazards regression models were adjusted for sex, age (<65, 65–75, or >75), year of operation (2000 to 2005, 2006 to 2010, or 2011 to 2015), tumor location (proximal colon, distal colon, or rectum), tumor–node–metastasis stage (I–II, III, or IV), tumor grade (well/moderately differentiated or poorly differentiated), lymphovascular invasion (no or yes), MMR status (proficient or deficient), *BRAF* status (wild-type or V600E mutant), and T-cell density score (low, intermediate, high, or missing). *P*_trend_ values were calculated by using the four ordinal categories of B-cell or plasma cell densities (Q1–Q4) as continuous variables in uni- and multivariable Cox proportional hazard regression models.

The prognostic role of different plasma cell and B-cell subsets largely reflected those of their main populations. High plasma cell densities were associated with improved CSS and OS in univariable Cox regression models, regardless of the specific subset (Supplementary Table S18). Interestingly, the IgG1^–^IgG2^–^ subset was associated with improved CSS in the CT (*P* = 0.0007) and IM (*P* = 0.0037) in the multivariable analyses, whereas IgG1^+^ plasma cells, typically considered to represent the main antitumor immunoglobulin, were associated with improved CSS only in the IM (*P* = 0.0002) but not in the CT (*P* = 0.010). High densities of several B-cell subsets were associated with longer CSS in the univariable Cox regression analyses (Supplementary Table S19), whereas in the multivariable models, only high IRF4^–^HLA-DR^–^ naïve B-cell density in the CT (*P* = 0.0017) remained independently associated with improved survival.

Given that high plasma cell densities in the CT were identified as an independent predictor of favorable outcomes, we explored the potential to combine this parameter with another immune cell parameter (densities of B cells, CD3^+^ T cells, CD8^+^ T cells, CD8^–^ T cells, or TLSs) to further enhance prognostic accuracy (Supplementary Tables S20 and S21; Supplementary Fig. S14). Among the parameters analyzed, the combination of plasma cell densities in the CT and TLS densities showed the strongest prognostic significance.

### Prognostically relevant plasma cell populations can be identified using a three-plex IHC assay

We tested whether the prognostic significance of plasma cells could be confirmed in an independent validation cohort (*n* = 737) by utilizing a three-plex IHC assay, a method more easily adaptable for clinical use. The associations of plasma cell and B-cell densities with the clinicopathologic characteristics in this cohort are shown in Supplementary Table S22. Similarly to the main cohort, high densities of both B cells and plasma cells in the CT and IM were associated with lower disease stage (*P* < 0.0001). Additionally, high plasma cell densities in both regions were associated with reduced lymphovascular invasion. Furthermore, both cell types in the IM also showed an association with MMR deficiency.

**Figure 3.**
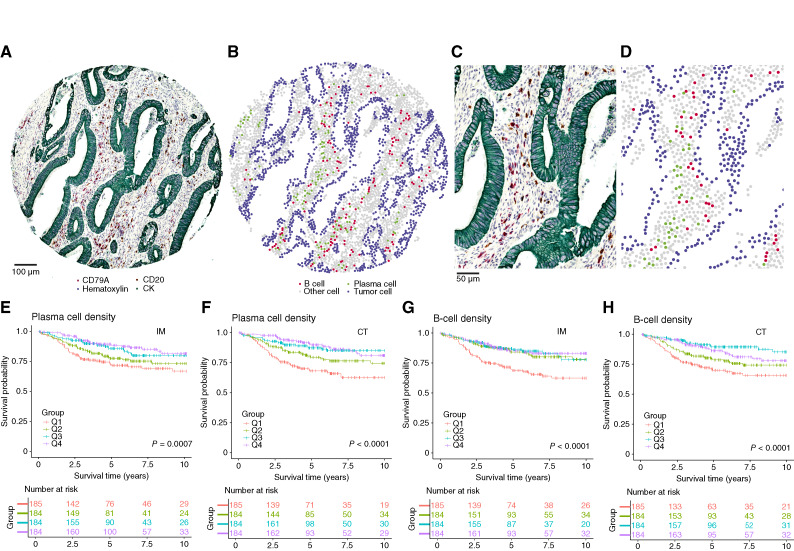
Three-plex IHC assay and Kaplan–Meier estimates of CSS in the validation cohort. The mIHC images (**A** and **C**) show representative example tumors, and the image analysis result images (**B** and **D**) show the main cell types identified. Kaplan–Meier curves visualize CSS according to the ordinal quartile categories (Q1–Q4, from low to high) of overall plasma cell densities in the IM (**E**) and CT (**F**) and overall B-cell densities in the IM (**G**) and CT (**H**). *P* values were calculated using the log-rank test.

In survival analysis, there were 252 deaths, including 139 cancer deaths, and the median follow-up time for censored cases was 5.9 years (IQR, 3.5–8.4). Kaplan–Meier analyses and univariable Cox regression models showed that high densities of both B and plasma cells in the CT and IM were associated with improved CSS (Fig. 3; Table 3). In multivariable Cox regression analyses, high plasma cell densities in the CT demonstrated a strong, independent association with prolonged CSS (HR for high vs. low 0.37, 95% CI, 0.21–0.65; *P*_trend_ = 0.0003), whereas B cells in neither the CT nor IM had statistically significant independent prognostic value. These results closely mirrored those of the main cohort, confirming that plasma cell densities in the CT harbor prognostic value independent of disease stage, MMR status, T-cell density score, and other factors. The three-plex IHC assay was also successfully applied to 30 whole-slide sections, showing moderate-to-good correlations with the densities observed in TMAs (Supplementary Fig. S15).

**Table 3.** Uni- and multivariable Cox regression models for CSS and OS according to overall plasma cell and B-cell densities in the CT and IM in the validation cohort.

		CSS			OS		
	No. of cases	No. of events	Univariable HR (95% CI)	Multivariable HR (95% CI)	No. of events	Univariable HR (95% CI)	Multivariable HR (95% CI)
Plasma cells							
CT							
Q1	185	58	1 (referent)	1 (referent)	79	1 (referent)	1 (referent)
Q2	184	37	0.59 (0.39–0.90)	0.75 (0.49–1.17)	66	0.74 (0.54–1.03)	0.85 (0.60–1.20)
Q3	184	23	0.35 (0.22–0.57)	0.56 (0.33–0.94)	51	0.55 (0.39–0.78)	0.73 (0.50–1.08)
Q4	184	21	0.32 (0.19–0.53)	0.37 (0.21–0.65)	56	0.60 (0.43–0.85)	0.65 (0.45–0.95)
*P*_trend_			<0.0001	0.0003		0.0009	0.019
IM							
Q1	185	50	1 (referent)	1 (referent)	73	1 (referent)	1 (referent)
Q2	184	39	0.75 (0.50–1.14)	1.17 (0.75–1.85)	65	0.87 (0.62–1.22)	1.04 (0.73–1.47)
Q3	184	27	0.50 (0.31–0.80)	1.07 (0.64–1.77)	56	0.72 (0.51–1.02)	1.09 (0.75–1.58)
Q4	184	23	0.41 (0.25–0.67)	1.01 (0.56–1.84)	58	0.69 (0.49–0.97)	1.04 (0.70–1.53)
*P*_trend_			<0.0001	0.95		0.019	0.79
B cells							
CT							
Q1	185	53	1 (referent)	1 (referent)	77	1 (referent)	1 (referent)
Q2	184	40	0.66 (0.44–1.00)	0.70 (0.45–1.08)	72	0.80 (0.58–1.11)	0.73 (0.52–1.02)
Q3	184	18	0.29 (0.17–0.50)	0.34 (0.19–0.61)	46	0.50 (0.34–0.71)	0.56 (0.38–0.82)
Q4	184	28	0.45 (0.29–0.71)	0.58 (0.33–1.01)	57	0.61 (0.43–0.85)	0.74 (0.50–1.11)
*P*_trend_			<0.0001	0.0057		0.0004	0.051
IM							
Q1	185	57	1 (referent)	1 (referent)	84	1 (referent)	1 (referent)
Q2	184	30	0.48 (0.31–0.75)	0.80 (0.49–1.29)	64	0.67 (0.49–0.94)	0.85 (0.60–1.22)
Q3	184	26	0.43 (0.27–0.69)	1.16 (0.69–1.95)	51	0.59 (0.41–0.83)	0.95 (0.64–1.39)
Q4	184	26	0.40 (0.25–0.64)	0.92 (0.50–1.68)	53	0.54 (0.39–0.77)	0.79 (0.51–1.20)
*P*_trend_			<0.0001	0.92		0.0003	0.38

Multivariable Cox proportional hazards regression models were adjusted for sex, age (<65, 65–75, >75), year of operation (2006–2010, 2011–2015, 2016–2020), tumor location (proximal colon, distal colon, rectum), tumor–node–metastasis stage (I–II, III, IV), tumor grade (well/moderately differentiated, poorly differentiated), lymphovascular invasion (no, yes), MMR status (proficient, deficient), *BRAF* status (wild-type, V600E mutant), and T-cell density score (low, intermediate, high). *P*_trend_ values were calculated by using the four ordinal categories of B-cell or plasma cell densities (Q1–Q4) as continuous variables in uni- and multivariable Cox proportional hazards regression models.

We analyzed the association between adjuvant treatment status and CSS across plasma cell densities in the CT in stages II and III. However, there was no statistically significant difference in the benefit associated with adjuvant chemotherapy among patients with high versus low plasma cell densities in either stage II or III (*P*_interaction_ > 0.24; Supplementary Fig. S16).

We also evaluated whether CD138 IHC alone could identify the prognostically relevant plasma cell population. However, CD138 stained plasma cells with variable intensity and frequently labeled tumor cells, complicating plasma cell identification (Supplementary Fig. S17). CD138^+^ plasma cell densities showed moderate correlation with CD79A^+^CD20^–^ plasma cell densities and a statistically nonsignificant trend toward favorable survival (Supplementary Fig. S18; Supplementary Table S23).

## Discussion

The aim of this study was to assess the significance of B-cell and plasma cell subsets in colorectal cancer. Using seven-plex IHC combined with machine learning–based image analysis on a large cohort of 912 patients with colorectal cancer, we found that high densities of CD20^–^CD79A^+^ plasma cells were associated with favorable disease outcomes independent of disease stage, MMR status, T-cell densities, and several other factors. This finding was validated in an independent cohort of 737 patients with colorectal cancer using a clinically applicable three-plex IHC assay. Spatial analyses revealed that plasma cells were located closer to tumor cells than B cells. Our study highlights the importance of different B-cell and plasma cell populations in colorectal cancer progression and suggests that plasma cell density measurements could complement the prognostic value of T-cell density measurements.

High plasma cell density in the CT was independently associated with improved CSS. To our knowledge, only one previous study has identified high plasma cell density as an independent prognostic factor for improved CSS (7). Other studies, however, have found no independent prognostic significance for plasma cells (21, 23). Many of these studies have utilized CD138 in plasma cell detection, which may partly explain the inconsistent results, as CD138 also labels some tumor cells and may be difficult to interpret (7, 23). Our study confirmed this, as CD138^+^ plasma cell densities showed a weaker association with patient outcomes than CD79A^+^CD20^–^ plasma cell densities. This supports the existing literature recommending CD79A and CD20 for plasma cell identification (23, 30). Based on the results from the main study cohort that highlighted the prognostic value of the general plasma cell population in the CT, we developed an additional chromogenic three-plex IHC assay for their identification. This assay is more easily adaptable to clinical workflows compared with the seven-plex IHC assay and could be automated on tissue stainers widely available in pathology laboratories worldwide.

The prognostic significance of plasma cells seemed relatively consistent across the subpopulations defined by IgG1 and IgG2 expression. Of the individual populations, the IgG1^–^IgG2^–^ subset showed the strongest association with improved survival, thus contradicting the hypothesized superior prognostic potential of the IgG1^+^ subset. In the literature, IgG1 is considered the main antitumor immunoglobulin due to its ability to trigger antibody-dependent cellular cytotoxicity, mediate complement-dependent cytotoxicity, and indirectly enhance antigen presentation to T lymphocytes (19). Besides IgG, the other known tumor-associated immunoglobulin isotypes are IgA and IgM (18). IgA is the predominant isotype in the gut of both healthy individuals and patients with colorectal cancer (39). Thus, the IgG1^–^IgG2^–^ subset may primarily consist of IgA^+^ plasma cells, and the prognostic role of the IgG1^–^IgG2^–^ subset might be related to the antitumor functions of IgA. For example, IgA has been shown to induce neutrophil-mediated tumor cell eradication more efficiently than IgG (40).

The prognostic role of B cells varied more across subpopulations compared with plasma cells. Although high densities of most B-cell subsets were associated with improved CSS in univariable Cox regression models, few demonstrated independent prognostic significance in multivariable models. This suggests that the antitumor functions of B cells may depend on interactions with other effector cells, particularly cytotoxic T cells, as CD20^+^ B cells have been shown to enhance cytotoxic T-cell antitumor activity in various cancers (22, 41, 42). This interplay could explain why most B-cell subsets lost independent prognostic value in multivariable models that included T-cell density scores. Conversely, T-cell densities showed weaker prognostic significance than plasma cell densities in the CT. A possible explanation is that T-cell densities are more strongly linked to MMR deficiency, which itself is associated with favorable survival and was included in multivariable Cox regression models.

The independent prognostic potential of high plasma cell density in the CT was further enhanced when analyzed alongside TLS density. As TLSs may serve as sites for B-cell differentiation into antibody-producing plasma cells (43, 44), such synergy can be expected. However, most colorectal cancer studies focusing on B cells and TLSs primarily utilize the pan–B-cell marker CD20 (9, 10), which does not label plasma cells (30). In other cancers, TLS-associated plasma cells typically represent either IgG or IgA phenotypes (44, 45), with IgG antibodies particularly showing a high affinity for tumor cells (45). Our results, however, indicate that the plasma cell population in colorectal cancer is largely composed of cells with a phenotype other than IgG. In addition, the correlation between high plasma cell density and TLS density was relatively weak, suggesting a synergistic rather than a strictly causal relationship between high plasma cell density and the established prognostic role of TLS density (9, 10). Further research is needed to clarify the interplay between TLSs and plasma cells in colorectal cancer.

There are some limitations to this study. First, it was carried out using TMAs instead of whole-tissue sections. Although TMAs have been criticized for representing only a small portion of each tumor, they enable an efficient analysis of large study cohorts. Furthermore, the results from whole-slide studies have been shown to be reproducible with a TMA-based approach (46), as confirmed by our analysis of 30 whole-slide sections. TMAs have also been successfully used to investigate the distribution of immune cell populations in the colorectal cancer microenvironment in numerous previous studies (7, 21, 22). The marker intensities were relatively consistent across the TMA sections, indicating successful immunostainings. Additionally, the staining patterns between standard IHC and mIHC were nearly identical, further validating the performance of the mIHC assay. Second, the image analysis was performed using a machine learning approach, which is inherently limited by the quality of algorithm training. Despite our rigorous optimization efforts, the analysis identified a small number of B cells and plasma cells with unlikely immunophenotypes (related to technical artifacts such as two overlapping cells). However, their proportion was minimal and likely did not affect our main findings. Despite certain technical limitations, machine learning–based approaches have been extensively used for the analysis of colorectal cancer immune infiltrates with promising results across various immune cell types (7, 8, 22, 25, 33). Such computer-assisted quantification can be used to produce reproducible data in IHC immune cell assays (14). In contrast, visual evaluation may be subject to greater interobserver variability (47). Third, this study focused on B cells and plasma cells, whereas several other relevant immune cell types, such as specific T helper cell subsets, were not analyzed. An integrated analysis of various immune cell types would enable the examination of cellular neighborhoods defined by multiple cell types (48) and provide deeper insights into their interactions and spatial localization. Furthermore, high-plex analyses such as spatial transcriptomics could offer potential mechanistic insights. Fourth, adjuvant treatment data were only available for the validation cohort, in which treatment selection was likely influenced by factors such as age, comorbidities, disease stage, and tumor characteristics. Therefore, although no significant difference was observed in the benefit of adjuvant chemotherapy among patients with high versus low plasma cell densities, further studies are needed to determine whether plasma cells could predict responses to specific treatments. One possible strategy is to integrate plasma cell density into artificial intelligence–based models designed to predict the response to certain treatments as shown by Foersch and colleagues (49). Fifth, recurrence-free survival data were not available. However, given the long follow-up duration, CSS should provide a reliable estimate of patient outcomes. Finally, *RAS* mutation status was also not available for either of our cohorts. Although it has been established as one of the key genetic factors of colorectal cancer, the prognostic significance of *RAS* mutation status is relatively weak in comparison with MMR status and *BRAF* status (50), which were available for both of our cohorts.

The strengths of the study include two relatively large colorectal cancer cohorts that have been extensively characterized for both histologic and molecular features (33, 37). The multivariable Cox regression models were adjusted for a comprehensive set of parameters, including MMR status and *BRAF* status, which are key molecular prognostic parameters of colorectal cancer. Additionally, the use of mIHC and machine learning–based image analysis allowed for precise immunophenotyping of B and plasma cells and uniform analysis of the cohorts. This approach also enabled spatial analyses to examine the localization of individual immune cells in the tumors, which are not possible using conventional qualitative methods. Finally, we applied a stringent statistical significance threshold, enhancing the reproducibility of our findings.

In conclusion, multiplexed, quantitative measurement of CD20^–^CD79A^+^ plasma cell densities in the colorectal cancer microenvironment represents a relevant tumor–immune biomarker that predicts survival independent of disease stage, MMR status, T-cell densities, and other clinicopathologic characteristics.

## Supplementary Material

Supplementary Tables and Figures1 Tables S1-S23 Figures S1-S18
