# Supplementary material for: Multimarker Assessment of B-Cell and Plasma Cell Subsets and Their Prognostic Role in the Colorectal Cancer Microenvironment
Source: Clin Cancer Res. 2025 Apr 8;31(12):2466–77. doi: 10.1158/1078-0432.CCR-24-4083 (PMC12163598; doi:10.1158/1078-0432.CCR-24-4083)
Supplement: Supplementary Tables and Figures1 — Tables S1-S23 Figures S1-S18 [file ccr-24-4083_supplementary_tables_and_figures1_suppsd1.pdf]

Table S1. Representativeness of the study participants.

Cancer type(s)/subtype(s)/stage(s)/condition	Colorectal cancer (CRC)
Consideration related to:	
Sex	Men are diagnosed with CRC more often than women (1). Between 2015 and 2019, the average annual incidence rate of CRC in the U.S. was 41.5 per 100,000 in men and 31.2 per 100,000 in women (2).
Age	CRC incidence increases with age (1–3), with the median age at diagnosis of approximately 70 years (3).
Race/ethnicity	The incidence and death rates of CRC differ across racial and ethnic groups. In the U.S., American Indian, Alaska Native, and Black populations experience the highest rates, while Asian American/Pacific Islander populations have the lowest (2).
Geography	CRC incidence is highest in Europe, Australia/New Zealand, Northern America, and Eastern Asia, while lowest rates are observed in Africa and South and Central Asia (1).
Other Considerations	In the U.S., the 5-year relative survival rate for CRC was 65% between 2012 and 2018 (2).
Overall representativeness of this study	The age distribution of our cohorts aligns with previously reported data. The male-to-female ratio was 1:1 in the study cohort and 1.12:1 in the validation cohort. Compared to global rates, female patients were slightly overrepresented in our cohorts. While ethnicity was not recorded, the patients were treated in two Finnish hospitals, where the majority of the population is non-Hispanic White. Further research is needed to confirm the applicability of these findings to patients of diverse ethnic backgrounds.

1. Bray F, Laversanne M, Sung H, Ferlay J, Siegel RL, Soerjomataram I, et al. Global cancer statistics 2022: GLOBOCAN estimates of incidence and mortality worldwide for 36 cancers in 185 countries. CA Cancer J Clin. 2024;74:229–63.
2. Siegel RL, Wagle NS, Cercek A, Smith RA, Jemal A. Colorectal cancer statistics, 2023. CA Cancer J Clin. 2023;73:233–54.
3. Sharma R, Abbasi-Kangevari M, Abd-Rabu R, Abidi H, Abu-Gharbieh E, Acuna JM, et al. Global, regional, and national burden of colorectal cancer and its risk factors, 1990–2019: a systematic analysis for the Global Burden of Disease Study 2019. Lancet Gastroenterol Hepatol. 2022;7:627–47.

Table S2. Multiplex immunohistochemistry staining panel used in the study.

Cycle	Marker	Clone	Host species	Manufacturer	Cat. number	Dilution	Antigen retrieval
1	CD20	L26	Mouse	Diagnostic Biosystems	Mob004	1:100	ER2, 20 min
2	CD79A	SP18	Rabbit	Thermo Fisher Scientific	RM-9118	1:600	ER1, 60 min
3	IRF4	MUM1p	Mouse	Dako	M7259	1:200	ER1, 60 min
4	IgG1	RM117	Rabbit	RevMAb	31-1019-00	1:4,000	ER1, 60 min
5	HLA-DR	TAL 1B5	Mouse	Santa Cruz	sc-53319	1:4,000	ER1, 60 min
6	IgG2	EPR4418	Rabbit	Abcam	ab134050	1:40,000	ER1, 60 min
7	IgG4	HP6025	Mouse	Thermo Fisher Scientific	605-900	1:500	ER1, 60 min
8	CK	BS5	Mouse	BioSite Histo	BSH-7124-1	1:400	ER1, 60 min

ER2: BOND Epitope Retrieval Solution 2 (Leica Biosystems, AR9640)
ER1: BOND Epitope Retrieval Solution 1 (Leica Biosystems, AR9961)

Table S3. Key resources utilized in this study.

Reagent or resource	Source	Identifier
**Antibodies**		
CD20	Diagnostic Biosystems	Mob004, RRID:AB_3676658
CD20	Leica Biosystems	CD20-L26-L-CE, RRID:AB_563520
CD79A	Thermo Fisher Scientific	RM-9118, RRID:AB_149967
CD79A	Cell Marque	179R-15, RRID:AB_3676661
CD138	Epredia	MS-1793-S, RRID:AB_149275
CK	BioSite Histo	BSH-7124-1, RRID:AB_3676662
CK	Leica Biosystems	AE1/AE3-601-L-CE, RRID:AB_2924990
HLA-DR	Santa Cruz	sc-53319, RRID:AB_629980
IgG1	RevMAb	31-1019-00, RRID:AB_2716359
IgG2	Abcam	ab134050, RRID:AB_3665956
IgG4	Thermo Fisher Scientific	605-900, RRID:AB_3676660
IRF4	Dako	M7259, RRID:AB_2127157
**Chemicals/ Immunohistochemistry reagents/ kits**		
BOND Epitope Retrieval Solution 1	Leica Biosystems	AR9961
BOND Epitope Retrieval Solution 2	Leica Biosystems	AR9640
BOND Dewax Solution	Leica Biosystems	AR9222
BOND Wash Solution	Leica Biosystems	AR9590
BOND Primary Antibody Diluent	Leica Biosystems	AR9352
BOND Polymer Refine Detection kit	Leica Biosystems	DS9800
ChromoPlex II Dual Detection kit	Leica Biosystems	DS9494
BOND Polymer HRP PLEX Detection	Leica Biosystems	DS9914
Green Chromogen	Leica Biosystems	DC9913
AEC^{+} high sensitivity substrate chromogen	Dako	K3469
VectaMount AQ	Vector Laboratories	H-5501
**Equipment**		
Leica BOND-III Stainer	Leica Biosystems	RRID:SCR_026521
Leica BOND RX Stainer	Leica Biosystems	RRID:SCR_025548
Leica Aperio AT2 Scanner	Leica Biosystems	RRID:SCR_021256
Hamamatsu NanoZoomer-XR	Hamamatsu Photonics	RRID:SCR_026520
**Software**		
R	R Core Team	RRID:SCR_001905
RStudio	RStudio Team	RRID:SCR_000432
QuPath	Bankhead P, et al. Sci Rep. 2017;7:16878.	RRID:SCR_018257
Fiji	Schindelin J, et al. Nat Methods. 2012;9:676–82.	RRID:SCR_002285

Table S4. Bake and dewax protocol.

Step	Reagent	Temperature	Duration
1	No reagent	60 °C	30 min
2	BOND Dewax Solution	72 °C	30 sec
3	BOND Dewax Solution	72 °C	0 min
4	BOND Dewax Solution	Ambient	0 min

This protocol was only performed before the first staining cycle.
BOND Dewax Solution (Leica Biosystems, AR9222)

Table S5. Heat-induced antigen retrieval protocol.

Step	Reagent	Temperature	Duration
1	BOND Epitope Retrieval Solution 1/2	Ambient	0 min
2	BOND Epitope Retrieval Solution 1/2	Ambient	0 min
3	BOND Epitope Retrieval Solution 1/2	100 °C	20–60 min
4	BOND Epitope Retrieval Solution 1/2	Ambient	0 min

This protocol was performed before each staining cycle.
BOND Epitope Retrieval Solution 1 (Leica Biosystems, AR9961)
BOND Epitope Retrieval Solution 2 (Leica Biosystems, AR9640)

Table S6. Protocol for the first immunostaining cycle with AEC^{+} chromogen.

Step(s)	Reagent	Duration
1	Peroxide Block[1]	5 min
2–4	BOND Wash Solution[2]	0 min
5	Antibody	30 min
6–8	BOND Wash Solution[2]	0 min
9	Post Primary[1]	8 min
10–11	BOND Wash Solution[2]	2 min
12	Polymer[1]	8 min
13–14	BOND Wash Solution[2]	2 min
15	H_2O	0 min
16	AEC[3]	0 min
17	AEC[3]	20 min
18–20	H_2O	0 min
21	Hematoxylin[1]	10 min
22	H_2O	0 min
23	BOND Wash Solution[2]	0 min
24	H_2O	0 min

[1]BOND Polymer Refine Detection kit (Leica Biosystems, DS9800)
[2]BOND Wash Solution (Leica Biosystems, AR9590)
[3]AEC^{+} high sensitivity substrate chromogen (Dako, K3469)

Table S7. Protocol for all subsequent mIHC staining cycles with AEC^+ chromogen and mouse antibodies.

Step(s)	Reagent	Duration
1–2	Alcohol	0 min
3	Alcohol	1 min
4–5	Alcohol	0 min
6–8	BOND Wash Solution[1]	0 min
9	BOND Wash Solution[1]	1 min
10–12	BOND Wash Solution[1]	0 min
13	Antibody	30 min
14–16	BOND Wash Solution[1]	0 min
17	Post Primary[2]	8 min
18–19	BOND Wash Solution[1]	2 min
20	Polymer[2]	8 min
21–22	BOND Wash Solution[1]	2 min
23	H_2O	0 min
24	AEC[3]	0 min
25	AEC[3]	20 min
26–28	H_2O	0 min
29	Hematoxylin[2]	10 min
30	H_2O	0 min
31	BOND Wash Solution[1]	0 min
32	H_2O	0 min

[1]BOND Wash Solution (Leica Biosystems, AR9590)
[2]BOND Polymer Refine Detection kit (Leica Biosystems, DS9800)
[3]AEC^+ high sensitivity substrate chromogen (Dako, K3469)

Table S8. Protocol for all subsequent mIHC staining cycles with AEC⁺ chromogen and rabbit antibodies.

Step(s)	Reagent	Duration
1–2	Alcohol	0 min
3	Alcohol	1 min
4–5	Alcohol	0 min
6–8	BOND Wash Solution[1]	0 min
9	BOND Wash Solution[1]	1 min
10–12	BOND Wash Solution[1]	0 min
13	Antibody	30 min
14	BOND Wash Solution[1]	0 min
15–16	BOND Wash Solution[1]	2 min
17	Polymer[2]	8 min
18–19	BOND Wash Solution[1]	2 min
20	H_2O	0 min
21	AEC[3]	0 min
22	AEC[3]	20 min
23–25	H_2O	0 min
26	Hematoxylin[2]	10 min
27	H_2O	0 min
28	BOND Wash Solution[1]	0 min
29	H_2O	0 min

[1]BOND Wash Solution (Leica Biosystems, AR9590)
[2]BOND Polymer Refine Detection kit (Leica Biosystems, DS9800)
[3]AEC$^+$ high sensitivity substrate chromogen (Dako, K3469)

Table S9. Functions, settings and parameters utilized in image analysis with QuPath software.

Function	Executing command	Used settings and parameters
Cell detection	Analyze → Cell detection → Cell detection	Default with following changes: Detection channel: Hematoxylin 1, Pixel size: 0.45 μm, Sigma: 1.4 μm, Minimum area: 15 $μm^2$, Cell expansion: 2.0 μm
Intensity features calculation	Analyze → Calculate features → Add intensity features	Default with following changes: Pixel size: 0.45 μm, Region: ROI, Tile diameter: 25 μm, Channel: Hematoxylin 1, Basic features: Mean + Standard deviation, Haralick features: default parameters
Smoothed features calculation	Analyze → Calculate features → Add smoothed features	Default settings and parameters
Pixel classification	Classify → Pixel classification → Train pixel classifier	Random trees (RTrees) classifier and following changes to the default Features settings: Channels: Hematoxylin 1 & 2 + cytokeratin, Scales: 1.0 and 4.0, sublevel Features: Gaussian, Laplacian of Gaussian, Weighed deviation and Gradient magnitude

Table S10. Immunophenotype characterization of the immune cell types utilized in this study.

Cohort	Immune cell type	Assay	Immunophenotype	Figures/Tables used
Jyväskylä (study)	B cells	7-plex IHC	$CD20^{+}CD79A^{+}$	Table: 1–2, S14–S16<br>Figure: 1–2, S8, S11–S12, S14
	Näive B cells	7-plex IHC	$CD20^{+}CD79A^{+}IRF4^{–}HLA\text{-}DR^{–}$	Table: S19<br>Figure: S5–S6, S8–S10
	Activated antigen-presenting B cells	7-plex IHC	$CD20^{+}CD79A^{+}IRF4^{–}HLA\text{-}DR^{+}$	Table: S19<br>Figure: S5–S6, S8–S10
	Memory B cells	7-plex IHC	$CD20^{+}CD79A^{+}IRF4^{+}HLA\text{-}DR^{+}$	Table: S19<br>Figure: S5–S6, S8–S10
	Plasma cells	7-plex IHC	$CD20^{–}CD79A^{+}$	Table: 1–2, S13, S15–S17, S20–S21<br>Figure: 1–2, S7, S11–S14
	$IgG1^{+}$ plasma cells	7-plex IHC	$CD20^{–}CD79A^{+}IRF4^{+}IgG1^{+}IgG2^{–}$	Table: S18<br>Figure: S5–S7, S9–S10
	$IgG2^{+}$ plasma cells	7-plex IHC	$CD20^{–}CD79A^{+}IRF4^{+}IgG1^{–}IgG2^{+}$	Table: S18<br>Figure: S5–S7, S9–S10
	Double-negative plasma cells	7-plex IHC	$CD20^{–}CD79A^{+}IRF4^{+}IgG1^{–}IgG2^{–}$	Table: S18<br>Figure: S5–S7, S9–S10
	T cells	Standard IHC*	$CD3^{+}$	Table: 2, S13–S19 as T cell density score and S20–S21 alone<br>Figure: S9, S14
	Cytotoxic T cells	Standard IHC*	$CD8^{+}$	Table: 2, S13–S19 as T cell density score and S20–S21 alone<br>Figure: S9, S14
	Helper T cells^	Standard IHC*	$CD3^{+}CD8^{–}$	Table: S20–S21<br>Figure: S9, S14
Oulu (validation)	B cells	3-plex mIHC	$CD20^{+}CD79A^{+}$	Table: 3, S22<br>Figure: 3, S15, S17
	Plasma cells	3-plex mIHC	$CD20^{–}CD79A^{+}$	Table: 3, S22<br>Figure: 3, S15–S18
	Plasma cells	Standard IHC	$CD138^{+}$ (for comparison)	Table: S23<br>Figure: S17–S18
	T cells	Standard IHC	$CD3^{+}$	Table: 3, S23 as T cell density score
	Cytotoxic T cells	Standard IHC	$CD8^{+}$	Table: 3, S23 as T cell density score

^Approximated by subtracting $CD8^{+}$ cytotoxic T cells from the total $CD3^{+}$ T cell population.
*Data from Elomaa *et al*. (Br J Cancer. 2022;127:514–523).

Table S11. Protocol for the three-plex immunohistochemistry assay.

Step(s)	Reagent	Temperature	Duration
1	BOND Epitope Retrieval Solution 2[1]	Ambient	0 min
2	BOND Epitope Retrieval Solution 2[1]	Ambient	0 min
3	BOND Epitope Retrieval Solution 2[1]	100 °C	30 min
4	BOND Epitope Retrieval Solution 2[1]	Ambient	0 min
5	BOND Wash Solution[2]	Ambient	0 min
6	Peroxide Block[3]	Ambient	5 min
7–9	BOND Wash Solution[2]	Ambient	0 min
10	Primary antibody (CD20+CD79A)[4]	Ambient	30 min
11–13	BOND Wash Solution[2]	Ambient	0 min
14	Polymer mHRP[3]	Ambient	8 min
15–18	BOND Wash Solution[2]	Ambient	0 min
19	Polymer rAP[3]	Ambient	8 min
20–23	BOND Wash Solution[2]	Ambient	0 min
24	H_2O	Ambient	0 min
25	DAB[3]	Ambient	0 min
26	DAB[3]	Ambient	10 min
27–29	H_2O	Ambient	0 min
30	Red Refine[3]	Ambient	10 min
31	Red Refine[3]	Ambient	5 min
32-34	H_2O	Ambient	0 min
35	BOND Wash Solution[2]	Ambient	0 min
36	H_2O	Ambient	0 min
37	BOND Epitope Retrieval Solution 1[5]	Ambient	0 min
38	BOND Epitope Retrieval Solution 1[5]	Ambient	0 min
39	BOND Epitope Retrieval Solution 1[5]	100 °C	30 min
40	BOND Epitope Retrieval Solution 1[5]	Ambient	0 min
41	Peroxide Block[6]	Ambient	5 min
42–44	BOND Wash Solution[2]	Ambient	0 min
45	Primary antibody (CK)[7]	Ambient	30 min
46–48	BOND Wash Solution[2]	Ambient	0 min
49	Post primary[6]	Ambient	8 min
50–53	BOND Wash Solution[2]	Ambient	2 min
54	Polymer HRP[6]	Ambient	8 min
55–58	BOND Wash Solution[2]	Ambient	2 min
60	Green chromogen[8]	Ambient	0 min
61	Green chromogen[8]	Ambient	5 min
62-65	H_2O	Ambient	0 min
66	Hematoxylin[3]	Ambient	5 min
67	H_2O	Ambient	0 min
68	BOND Wash Solution[2]	Ambient	0 min
69	H_2O	Ambient	0 min

[1]BOND Epitope Retrieval Solution 2 (Leica Biosystems, AR9640)
[2]BOND Wash Solution (Leica Biosystems, AR9590)
[3]ChromoPlex II Dual Detection kit (Leica Biosystems, DS9494)
[4]CD20 (Leica Biosystems, CD20-L26-L-CE, L26, 1:75) and CD79A (Cell Marque, 179R-15, SP18, 1:800) antibodies diluted in BOND Primary Antibody Diluent (Leica Biosystems, AR9352)
[5]BOND Epitope Retrieval Solution 1 (Leica Biosystems, AR9961)
[6]BOND Polymer HRP PLEX Detection (Leica Biosystems, DS9914)
[7]CK (Leica Biosystems, AE1/AE3-601-L-CE, AE1/AE3, 1:55) antibodies diluted in BOND Primary Antibody Diluent (Leica Biosystems, AR9352)
[8]Green Chromogen (Leica Biosystems, DC9913)

Table S12. Protocol for CD138 immunohistochemistry

Step(s)	Reagent	Duration
1	Peroxide Block[1]	5 min
2–4	BOND Wash Solution[2]	0 min
5	Antibody[3]	30 min
6–8	BOND Wash Solution[2]	0 min
9	Post Primary[1]	8 min
10–11	BOND Wash Solution[2]	2 min
12	Polymer[1]	8 min
13–14	BOND Wash Solution[2]	2 min
15	H_2O	0 min
16	DAB[1]	0 min
17	DAB[1]	10 min
18–20	H_2O	0 min
21	Hematoxylin[1]	10 min
22	H_2O	0 min
23	BOND Wash Solution[2]	0 min
24	H_2O	0 min

[1]BOND Polymer Refine Detection kit (Leica Biosystems, DS9800)
[2]BOND Wash Solution (Leica Biosystems, AR9590)
[3]Clone MI15, 1:50 (Epredia MS-1793-S)
The protocol was preceded by bake and dewax (**Table S4**) and antigen retrieval (**Table S5**).

Table S13. Multivariable Cox regression models for cancer-specific survival and overall survival according to plasma cell densities.

Variable	N	Overall plasma cell density, CT		Overall plasma cell density, IM	
		Cancer-specific survival	Overall survival	Cancer-specific survival	Overall survival
		Multivariable HR (95% CI)	Multivariable HR (95% CI)	Multivariable HR (95% CI)	Multivariable HR (95% CI)
Plasma cell density					
Q1	228	1 (referent)	1 (referent)	1 (referent)	1 (referent)
Q2	228	0.66 (0.47–0.93)	0.69 (0.53–0.90)	1.04 (0.76–1.44)	1.07 (0.83–1.37)
Q3	228	0.64 (0.45–0.93)	0.73 (0.56–0.96)	0.66 (0.46–0.97)	0.81 (0.62–1.06)
Q4	228	0.48 (0.32–0.72)	0.65 (0.49–0.87)	0.67 (0.45–1.00)	0.78 (0.59–1.03)
Gender					
Male	456	1 (referent)	1 (referent)	1 (referent)	1 (referent)
Female	456	0.84 (0.65–1.10)	0.70 (0.58–0.85)	0.81 (0.62–1.06)	0.69 (0.57–0.84)
Age					
<65	245	1 (referent)	1 (referent)	1 (referent)	1 (referent)
65–75	328	1.01 (0.73–1.40)	1.24 (0.95–1.62)	1.08 (0.78–1.49)	1.28 (0.98–1.67)
>75	339	1.84 (1.34–2.54)	3.03 (2.36–3.89)	1.83 (1.33–2.52)	3.00 (2.34–3.86)
Year of operation					
2000–2005	271	1 (referent)	1 (referent)	1 (referent)	1 (referent)
2006–2010	295	0.67 (0.49–0.90)	0.68 (0.54–0.85)	0.67 (0.49–0.90)	0.69 (0.55–0.86)
2011–2015	346	0.44 (0.32–0.61)	0.55 (0.43–0.70)	0.45 (0.33–0.63)	0.55 (0.44–0.70)
Tumor location					
Proximal colon	448	1 (referent)	1 (referent)	1 (referent)	1 (referent)
Distal colon	336	0.74 (0.55–1.00)	0.87 (0.70–1.09)	0.77 (0.58–1.04)	0.89 (0.72–1.11)
Rectum	128	0.75 (0.50–1.13)	0.86 (0.63–1.16)	0.73 (0.49–1.10)	0.84 (0.62–1.13)
AJCC disease stage					
I–II	490	1 (referent)	1 (referent)	1 (referent)	1 (referent)
III	306	2.83 (2.01–3.99)	1.41 (1.13–1.77)	2.74 (1.94–3.86)	1.38 (1.10–1.72)
IV	116	15.7 (10.9–22.7)	7.20 (5.51–9.41)	15.0 (10.4–21.7)	6.92 (5.29–9.04)
Tumor differentiation					
Well to moderate (1–2)	760	1 (referent)	1 (referent)	1 (referent)	1 (referent)
Poor (3)	152	1.97 (1.40–2.78)	2.10 (1.62–2.72)	2.07 (1.47–2.91)	2.18 (1.69–2.82)
Lymphovascular invasion					
No	717	1 (referent)	1 (referent)	1 (referent)	1 (referent)
Yes	195	1.94 (1.47–2.55)	1.57 (1.26–1.96)	1.99 (1.51–2.61)	1.58 (1.27–1.97)
MMR status					
MMR proficient	771	1 (referent)	1 (referent)	1 (referent)	1 (referent)
MMR deficient	141	0.53 (0.31–0.93)	0.65 (0.45–0.95)	0.58 (0.34–1.01)	0.69 (0.48–1.01)
BRAF mutation					
Wild-type	759	1 (referent)	1 (referent)	1 (referent)	1 (referent)
V600E mutant	153	1.22 (0.77–1.93)	1.39 (1.00–1.95)	1.20 (0.76–1.89)	1.36 (0.98–1.89)
T cell density score					
Low	141	1 (referent)	1 (referent)	1 (referent)	1 (referent)
Intermediate	530	0.99 (0.71–1.39)	0.90 (0.70–1.17)	0.91 (0.65–1.26)	0.84 (0.65–1.09)
High	207	0.77 (0.47–1.27)	0.77 (0.54–1.10)	0.66 (0.41–1.06)	0.70 (0.51–0.98)
Missing	34	1.33 (0.70–2.54)	0.84 (0.49–1.45)	1.24 (0.65–2.35)	0.79 (0.46–1.35)

CI = Confidence interval, HR = Hazard ratio.

Multivariable Cox proportional hazards regression models were adjusted for sex, age (<65, 65–75, >75), year of operation (2000–2005, 2006–2010, 2011–2015), tumor location (proximal colon, distal colon, rectum), TNM stage (I–II, III, IV), tumor grade (well/moderately differentiated, poorly differentiated), lymphovascular invasion (no, yes), MMR status (proficient, deficient), *BRAF* status (wild-type, V600E mutant), and T cell density score (low, intermediate, high, missing).

Table S14. Multivariable Cox regression models for cancer-specific survival and overall survival according to B cell densities.

Variable	N	Overall B cell density, CT		Overall B cell density, IM	
		Cancer-specific survival	Overall survival	Cancer-specific survival	Overall survival
		Multivariable HR (95% CI)	Multivariable HR (95% CI)	Multivariable HR (95% CI)	Multivariable HR (95% CI)
B cell density					
Q1	228	1 (referent)	1 (referent)	1 (referent)	1 (referent)
Q2	228	0.72 (0.50–1.03)	0.77 (0.59–1.00)	0.96 (0.69–1.32)	0.92 (0.72–1.19)
Q3	228	0.75 (0.53–1.07)	0.84 (0.64–1.09)	0.65 (0.44–0.96)	0.85 (0.64–1.12)
Q4	228	0.71 (0.49–1.04)	0.84 (0.64–1.10)	0.73 (0.49–1.08)	0.88 (0.66–1.17)
Gender					
Male	456	1 (referent)	1 (referent)	1 (referent)	1 (referent)
Female	456	0.83 (0.64–1.09)	0.70 (0.58–0.86)	0.81 (0.62–1.05)	0.70 (0.57–0.85)
Age					
<65	245	1 (referent)	1 (referent)	1 (referent)	1 (referent)
65–75	328	1.08 (0.79–1.50)	1.29 (0.99–1.68)	1.10 (0.80–1.53)	1.28 (0.98–1.68)
>75	339	1.88 (1.36–2.59)	3.05 (2.37–3.93)	1.83 (1.32–2.53)	3.02 (2.35–3.89)
Year of operation					
2000–2005	271	1 (referent)	1 (referent)	1 (referent)	1 (referent)
2006–2010	295	0.66 (0.49–0.90)	0.68 (0.54–0.85)	0.65 (0.48–0.88)	0.68 (0.54–0.85)
2011–2015	346	0.47 (0.34–0.64)	0.56 (0.45–0.71)	0.46 (0.33–0.63)	0.56 (0.44–0.71)
Tumor location					
Proximal colon	448	1 (referent)	1 (referent)	1 (referent)	1 (referent)
Distal colon	336	0.78 (0.58–1.05)	0.90 (0.72–1.12)	0.79 (0.59–1.06)	0.91 (0.73–1.13)
Rectum	128	0.70 (0.46–1.05)	0.82 (0.60–1.11)	0.72 (0.48–1.08)	0.84 (0.62–1.13)
AJCC disease stage					
I–II	490	1 (referent)	1 (referent)	1 (referent)	1 (referent)
III	306	2.76 (1.95–3.90)	1.38 (1.10–1.72)	2.75 (1.95–3.88)	1.39 (1.11–1.73)
IV	116	15.9 (11.0–23.1)	7.21 (5.51–9.43)	14.9 (10.3–21.6)	7.06 (5.39–9.24)
Tumor differentiation					
Well to moderate (1–2)	760	1 (referent)	1 (referent)	1 (referent)	1 (referent)
Poor (3)	152	1.90 (1.35–2.68)	2.06 (1.59–2.67)	2.13 (1.51–3.01)	2.17 (1.68–2.82)
Lymphovascular invasion					
No	717	1 (referent)	1 (referent)	1 (referent)	1 (referent)
Yes	195	1.92 (1.45–2.53)	1.57 (1.26–1.96)	2.01 (1.53–2.64)	1.59 (1.27–1.98)
MMR status					
MMR proficient	771	1 (referent)	1 (referent)	1 (referent)	1 (referent)
MMR deficient	141	0.56 (0.32–0.97)	0.67 (0.46–0.97)	0.58 (0.33–1.01)	0.68 (0.47–0.99)
BRAF mutation					
Wild-type	759	1 (referent)	1 (referent)	1 (referent)	1 (referent)
V600E mutant	153	1.24 (0.78–1.96)	1.39 (0.99–1.94)	1.26 (0.80–1.99)	1.40 (1.00–1.95)
T cell density score					
Low	141	1 (referent)	1 (referent)	1 (referent)	1 (referent)
Intermediate	530	0.88 (0.63–1.23)	0.82 (0.64–1.06)	0.89 (0.64–1.24)	0.83 (0.64–1.07)
High	207	0.64 (0.39–1.04)	0.67 (0.48–0.94)	0.64 (0.40–1.03)	0.67 (0.48–0.94)
Missing	34	1.13 (0.59–2.16)	0.76 (0.44–1.30)	1.19 (0.62–2.28)	0.76 (0.44–1.30)

CI = Confidence interval, HR = Hazard ratio.

Multivariable Cox proportional hazards regression models were adjusted for sex, age (<65, 65–75, >75), year of operation (2000–2005, 2006–2010, 2011–2015), tumor location (proximal colon, distal colon, rectum), TNM stage (I–II, III, IV), tumor grade (well/moderately differentiated, poorly differentiated), lymphovascular invasion (no, yes), MMR status (proficient, deficient), *BRAF* status (wild-type, V600E mutant), and T cell density score (low, intermediate, high, missing).

Table S15. Univariable and multivariable Cox regression models for cancer-specific survival and overall survival according to tumor stromal plasma cell and B cell densities in the tumor center and invasive margin.

		Colorectal cancer-specific survival			Overall survival		
	No. of cases	No. of events	Univariable HR (95% CI)	Multivariable HR (95% CI)	No. of events	Univariable HR (95% CI)	Multivariable HR (95% CI)
Plasma cells							
Tumor center							
Q1	228	89	1 (referent)	1 (referent)	137	1 (referent)	1 (referent)
Q2	228	61	0.60 (0.43-0.83)	0.64 (0.45-0.90)	111	0.69 (0.54-0.88)	0.72 (0.55-0.94)
Q3	228	63	0.63 (0.46-0.87)	0.77 (0.53-1.10)	116	0.73 (0.57-0.93)	0.82 (0.62-1.07)
Q4	228	35	0.32 (0.22-0.48)	0.46 (0.30-0.72)	91	0.52 (0.40-0.68)	0.64 (0.48-0.86)
P_{trend}			<0.0001	0.0026		<0.0001	0.014
Invasive margin							
Q1	228	82	1 (referent)	1 (referent)	130	1 (referent)	1 (referent)
Q2	228	68	0.81 (0.59-1.12)	0.80 (0.57-1.13)	121	0.91 (0.71-1.16)	0.90 (0.70-1.17)
Q3	228	56	0.63 (0.45-0.88)	0.79 (0.56-1.14)	104	0.71 (0.55-0.92)	0.81 (0.62-1.07)
Q4	228	42	0.45 (0.31-0.66)	0.62 (0.42-0.92)	100	0.66 (0.51-0.85)	0.77 (0.58-1.01)
P_{trend}			<0.0001	0.022		0.0003	0.047
B cells							
Tumor center							
Q1	228	84	1 (referent)	1 (referent)	137	1 (referent)	1 (referent)
Q2	228	58	0.60 (0.43-0.84)	0.77 (0.54-1.10)	97	0.60 (0.46-0.78)	0.70 (0.53-0.92)
Q3	228	57	0.60 (0.43-0.84)	0.65 (0.45-0.93)	118	0.75 (0.59-0.96)	0.78 (0.60-1.01)
Q4	228	49	0.52 (0.37-0.75)	0.64 (0.44-0.94)	103	0.66 (0.51-0.85)	0.76 (0.58-1.00)
P_{trend}			0.0004	0.012		0.011	0.093
Invasive margin							
Q1	228	83	1 (referent)	1 (referent)	129	1 (referent)	1 (referent)
Q2	228	71	0.82 (0.60-1.13)	0.93 (0.67-1.28)	118	0.88 (0.68-1.13)	0.90 (0.69-1.16)
Q3	228	48	0.55 (0.39-0.78)	0.73 (0.50-1.07)	104	0.75 (0.58-0.97)	0.86 (0.65-1.13)
Q4	228	46	0.52 (0.36-0.74)	0.70 (0.47-1.05)	104	0.74 (0.57-0.95)	0.93 (0.70-1.23)
P_{trend}			<0.0001	0.044		0.0093	0.53

CI = Confidence interval, HR = Hazard ratio.

Multivariable Cox proportional hazards regression models were adjusted for sex, age (<65, 65–75, >75), year of operation (2000–2005, 2006–2010, 2011–2015), tumor location (proximal colon, distal colon, rectum), TNM stage (I–II, III, IV), tumor grade (well/moderately differentiated, poorly differentiated), lymphovascular invasion (no, yes), MMR status (proficient, deficient), *BRAF* status (wild-type, V600E mutant), and T cell density score (low, intermediate, high, missing). P_{trend} values were calculated by using the four ordinal categories of B cell or plasma cell densities (Q1–Q4) as continuous variables in univariable and multivariable Cox proportional hazard regression models.

Table S16. Univariable and multivariable Cox regression models for cancer-specific survival and overall survival according to overall plasma cell and B cell densities as two-category variables in the study cohort.

		Colorectal cancer-specific survival			Overall survival		
	No. of cases	No. of events	Univariable HR (95% CI)	Multivariable HR (95% CI)	No. of events	Univariable HR (95% CI)	Multivariable HR (95% CI)
Plasma cells							
Tumor center							
Low (Q1–Q2)	456	149	1 (referent)	1 (referent)	245	1 (referent)	1 (referent)
High (Q3–Q4)	456	99	0.62 (0.48–0.79)	0.65 (0.49–0.85)	210	0.77 (0.64–0.93)	0.82 (0.67–1.00)
P			0.0002	0.0020		0.0066	0.047
Invasive margin							
Low (Q1–Q2)	456	156	1 (referent)	1 (referent)	254	1 (referent)	1 (referent)
High (Q3–Q4)	456	92	0.53 (0.41–0.68)	0.57 (0.43–0.75)	201	0.68 (0.57–0.82)	0.70 (0.57–0.85)
P			<0.0001	<0.0001		<0.0001	0.0003
B cells							
Tumor center							
Low (Q1–Q2)	456	135	1 (referent)	1 (referent)	236	1 (referent)	1 (referent)
High (Q3–Q4)	456	113	0.80 (0.62–1.03)	0.96 (0.74–1.25)	219	0.89 (0.74–1.07)	1.02 (0.84–1.24)
P			0.078	0.76		0.21	0.81
Invasive margin							
Low (Q1–Q2)	456	156	1 (referent)	1 (referent)	248	1 (referent)	1 (referent)
High (Q3–Q4)	456	92	0.56 (0.43–0.72)	0.58 (0.44–0.77)	207	0.77 (0.64–0.93)	0.79 (0.65–0.97)
P			<0.0001	0.0002		0.0061	0.026

CI = Confidence interval, HR = Hazard ratio.

Multivariable Cox proportional hazards regression models were adjusted for sex, age (<65, 65–75, >75), year of operation (2000–2005, 2006–2010, 2011–2015), tumor location (proximal colon, distal colon, rectum), TNM stage (I–II, III, IV), tumor grade (well/moderately differentiated, poorly differentiated), lymphovascular invasion (no, yes), MMR status (proficient, deficient), *BRAF* status (wild-type, V600E mutant), and T cell density score (low, intermediate, high, missing).

Table S17. Univariable and multivariable Cox regression models for cancer-specific and overall survival according to plasma cell densities in the tumor center in various TNM stages in the study cohort.

		Colorectal cancer-specific survival			Overall survival		
	No. of cases	No. of events	Univariable HR (95% CI)	Multivariable HR (95% CI)	No. of events	Univariable HR (95% CI)	Multivariable HR (95% CI)
**T1–2**	186						
Plasma cell tumor center							
Low (Q1–Q2)	75	10	1 (referent)	1 (referent)	31	1 (referent)	1 (referent)
High (Q3–Q4)	111	7	0.44 (0.17–1.16)	0.46 (0.15–1.47)	32	0.63 (0.39–1.04)	0.56 (0.31–1.01)
P			0.10	0.19		0.071	0.054
**T3–4**	726						
Plasma cell tumor center							
Low (Q1–Q2)	381	139	1 (referent)	1 (referent)	214	1 (referent)	1 (referent)
High (Q3–Q4)	345	92	0.69 (0.53–0.90)	0.66 (0.50–0.88)	178	0.85 (0.69–1.03)	0.84 (0.68–1.04)
P			0.0055	0.0048		0.098	0.11
$P_{interaction}$			0.40	0.77		0.33	0.65
**N0**	516						
Plasma cell tumor center							
Low (Q1–Q2)	238	40	1 (referent)	1 (referent)	238	1 (referent)	1 (referent)
High (Q3–Q4)	278	32	0.65 (0.41–1.04)	0.76 (0.46–1.27)	278	0.85 (0.65–1.12)	0.93 (0.69–1.24)
P			0.074	0.30		0.25	0.61
**N1–2**	396						
Plasma cell tumor center							
Low (Q1–Q2)	218	109	1 (referent)	1 (referent)	141	1 (referent)	1 (referent)
High (Q3–Q4)	178	67	0.70 (0.51–0.94)	0.64 (0.45–0.90)	100	0.78 (0.61–1.01)	0.73 (0.55–0.97)
P			0.019	0.010		0.064	0.029
$P_{interaction}$			0.88	0.77		0.52	0.24
**M0**	796						
Plasma cell tumor center							
Low (Q1–Q2)	390	93	1 (referent)	1 (referent)	181	1 (referent)	1 (referent)
High (Q3–Q4)	406	61	0.60 (0.43–0.82)	0.62 (0.44–0.87)	166	0.82 (0.66–1.01)	0.85 (0.67–1.06)
P			0.0017	0.0063		0.060	0.14
**M1**	116						
Plasma cell tumor center							
Low (Q1–Q2)	66	56	1 (referent)	1 (referent)	64	1 (referent)	1 (referent)
High (Q3–Q4)	50	38	0.70 (0.46–1.06)	0.80 (0.48–1.34)	50	0.68 (0.46–1.01)	0.85 (0.53–1.37)
P			0.093	0.40		0.056	0.51
$P_{interaction}$			0.67	0.13		0.28	0.87

CI = Confidence interval, HR = Hazard ratio.

Multivariable Cox proportional hazards regression models were adjusted for sex, age (<65, 65–75, >75), year of operation (2000–2005, 2006–2010, 2011–2015), tumor location (proximal colon, distal colon, rectum), tumor grade (well/moderately differentiated, poorly differentiated), lymphovascular invasion (no, yes), MMR status (proficient, deficient), *BRAF* status (wild-type, V600E mutant), and T cell density score (low, intermediate, high, missing). $P_{interaction}$ was calculated using the Wald test for the cross product of plasma cell density (low vs. high) and AJCC stage variables (T1–2 vs. T3–4; N0 vs. N1–2; M0 vs. M1) in the Cox regression model.

Table S18. Univariable and multivariable Cox regression models for cancer-specific and overall survival according to overall densities of IgG1$^{+/–}$, IgG2$^{+/–}$, and IgG1$^{–}$IgG2$^{–}$ plasma cells in the tumor center and invasive margin.

		Colorectal cancer-specific survival			Overall survival		
	No. of cases	No. of events	Univariable HR (95% CI)	Multivariable HR (95% CI)	No. of events	Univariable HR (95% CI)	Multivariable HR (95% CI)
IgG1^{+} plasma cells							
Tumor center							
Q1	228	89	1 (referent)	1 (referent)	136	1 (referent)	1 (referent)
Q2	228	59	0.56 (0.41–0.78)	0.72 (0.51–1.00)	115	0.72 (0.56–0.92)	0.84 (0.65–1.09)
Q3	228	59	0.58 (0.42–0.80)	0.73 (0.51–1.05)	104	0.67 (0.52–0.86)	0.79 (0.60–1.03)
Q4	228	41	0.39 (0.27–0.57)	0.57 (0.38–0.87)	100	0.62 (0.48–0.80)	0.76 (0.57–1.01)
P_{trend}			<0.0001	0.010		0.0003	0.054
Invasive margin							
Q1	228	91	1 (referent)	1 (referent)	142	1 (referent)	1 (referent)
Q2	228	62	0.62 (0.45–0.86)	0.72 (0.52–1.01)	111	0.71 (0.55–0.91)	0.77 (0.59–0.99)
Q3	228	58	0.56 (0.41–0.78)	0.62 (0.44–0.88)	110	0.67 (0.53–0.86)	0.72 (0.56–0.94)
Q4	228	37	0.35 (0.24–0.51)	0.49 (0.33–0.74)	92	0.54 (0.41–0.70)	0.69 (0.52–0.91)
P_{trend}			<0.0001	0.0002		<0.0001	0.0074
IgG1$^{–}$ plasma cells							
Tumor center							
Q1	228	85	1 (referent)	1 (referent)	137	1 (referent)	1 (referent)
Q2	228	61	0.63 (0.46–0.88)	0.62 (0.44–0.89)	105	0.66 (0.51–0.85)	0.65 (0.50–0.85)
Q3	228	62	0.66 (0.48–0.92)	0.71 (0.50–1.00)	114	0.73 (0.57–0.94)	0.76 (0.59–0.99)
Q4	228	40	0.40 (0.27–0.58)	0.44 (0.29–0.67)	99	0.59 (0.46–0.77)	0.63 (0.47–0.84)
P_{trend}			<0.0001	0.0007		0.0004	0.010
Invasive margin							
Q1	228	78	1 (referent)	1 (referent)	126	1 (referent)	1 (referent)
Q2	228	75	0.96 (0.70–1.31)	0.99 (0.71–1.36)	130	1.03 (0.81–1.32)	1.05 (0.82–1.35)
Q3	228	54	0.62 (0.44–0.88)	0.74 (0.51–1.06)	101	0.70 (0.54–0.91)	0.79 (0.60–1.03)
Q4	228	41	0.46 (0.31–0.67)	0.59 (0.39–0.88)	98	0.65 (0.50–0.85)	0.73 (0.55–0.96)
P_{trend}			<0.0001	0.0041		<0.0001	0.0077
IgG2^{+} plasma cells							
Tumor center							
Q1	228	79	1 (referent)	1 (referent)	129	1 (referent)	1 (referent)
Q2	228	64	0.72 (0.52–1.00)	0.74 (0.52–1.03)	122	0.83 (0.65–1.06)	0.83 (0.64–1.07)
Q3	228	62	0.69 (0.49–0.96)	0.76 (0.54–1.07)	106	0.70 (0.54–0.91)	0.78 (0.60–1.02)
Q4	228	43	0.48 (0.33–0.69)	0.64 (0.43–0.95)	98	0.65 (0.50–0.85)	0.78 (0.59–1.03)
P_{trend}			0.0001	0.032		0.0006	0.066
Invasive margin							
Q1	228	77	1 (referent)	1 (referent)	129	1 (referent)	1 (referent)
Q2	228	71	0.87 (0.63–1.20)	0.88 (0.63–1.23)	120	0.88 (0.68–1.12)	0.85 (0.66–1.10)
Q3	228	55	0.65 (0.46–0.92)	0.77 (0.54–1.11)	106	0.74 (0.57–0.95)	0.85 (0.65–1.11)
Q4	228	45	0.53 (0.36–0.76)	0.75 (0.51–1-10)	100	0.68 (0.57–0.88)	0.84 (0.64–1.10)
P_{trend}			0.0002	0.097		0.0015	0.21
IgG2$^{–}$ plasma cells							
Tumor center							
Q1	228	85	1 (referent)	1 (referent)	136	1 (referent)	1 (referent)
Q2	228	63	0.66 (0.48–0.92)	0.65 (0.46–0.92)	110	0.70 (0.55–0.91)	0.71 (0.55–0.92)
Q3	228	60	0.63 (0.45–0.87)	0.68 (0.47–0.97)	109	0.69 (0.53–0.88)	0.74 (0.56–0.96)
Q4	228	40	0.41 (0.28–0.59)	0.44 (0.29–0.67)	100	0.61 (0.47–0.79)	0.66 (0.49–0.88)
P_{trend}			<0.0001	0.0003		0.0003	0.0080
Invasive margin							
Q1	228	77	1 (referent)	1 (referent)	124	1 (referent)	1 (referent)
Q2	228	78	1.02 (0.74–1.40)	1.01 (0.74–1.40)	130	1.06 (0.83–1.36)	1.07 (0.83–1.38)
Q3	228	53	0.63 (0.44–0.89)	0.74 (0.51–1.07)	103	0.74 (0.57–0.96)	0.84 (0.64–1.10)
Q4	228	40	0.46 (0.31–0.67)	0.64 (0.43–0.97)	98	0.68 (0.52–0.88)	0.81 (0.61–1.07)
P_{trend}			<0.0001	0.014		0.0003	0.057

(continued)

IgG1⁻IgG2⁻ plasma cells							
Tumor center							
Q1	228	84	1 (referent)	1 (referent)	137	1 (referent)	1 (referent)
Q2	228	60	0.63 (0.45–0.88)	0.61 (0.43–0.87)	105	0.67 (0.52–0.86)	0.69 (0.53–0.91)
Q3	228	62	0.66 (0.47–0.91)	0.70 (0.49–1.00)	109	0.69 (0.53–0.88)	0.75 (0.57–0.97)
Q4	228	42	0.43 (0.30–0.62)	0.45 (0.30–0.68)	104	0.63 (0.49–0.82)	0.66 (0.50–0.88)
P_{trend}			<0.0001	0.0007		0.0010	0.012
Invasive margin							
Q1	228	76	1 (referent)	1 (referent)	124	1 (referent)	1 (referent)
Q2	228	77	1.00 (0.73–1.37)	0.90 (0.64–1.25)	131	1.05 (0.82–1.35)	0.96 (0.74–1.24)
Q3	228	54	0.64 (0.45–0.91)	0.70 (0.48–1.02)	102	0.72 (0.55–0.94)	0.76 (0.58–1.00)
Q4	228	41	0.47 (0.32–0.69)	0.58 (0.39–0.87)	98	0.67 (0.51–0.87)	0.73 (0.55–0.97)
P_{trend}			<0.0001	0.0037		0.0002	0.010

CI = Confidence interval, HR = Hazard ratio.

Multivariable Cox proportional hazards regression models were adjusted for sex, age (<65, 65–75, >75), year of operation (2000–2005, 2006–2010, 2011–2015), tumor location (proximal colon, distal colon, rectum), TNM stage (I–II, III, IV), tumor grade (well/moderately differentiated, poorly differentiated), lymphovascular invasion (no, yes), MMR status (proficient, deficient), *BRAF* status (wild-type, V600E mutant), and T cell density score (low, intermediate, high, missing). P_{trend} values were calculated by using the four ordinal categories of plasma cell densities (Q1–Q4) as continuous variables in univariable and multivariable Cox proportional hazard regression models.

Table S19. Univariable and multivariable Cox regression models for cancer-specific and overall survival according to overall densities of IRF4$^{+/-}$, HLA-DR$^{+/-}$, IRF4^{+}HLA-DR^{+}, IRF4^{-}HLA-DR^{+}, and IRF4^{-}HLA-DR^{-} B cells in the tumor center and invasive margin.

		Colorectal cancer-specific survival			Overall survival		
	No. of cases	No. of events	Univariable HR (95% CI)	Multivariable HR (95% CI)	No. of events	Univariable HR (95% CI)	Multivariable HR (95% CI)
IRF4^{+} B cells							
Tumor center							
Q1	320	109	1 (referent)	1 (referent)	168	1 (referent)	1 (referent)
Q2	197	52	0.72 (0.52–1.00)	0.65 (0.46–0.92)	103	0.92 (0.72–1.18)	0.88 (0.68–1.14)
Q3	197	51	0.72 (0.52–1.00)	0.80 (0.57–1.14)	101	0.92 (0.72–1.17)	1.00 (0.77–1.29)
Q4	198	36	0.50 (0.34–0.73)	0.62 (0.41–0.93)	83	0.74 (0.57–0.97)	0.86 (0.65–1.14)
P_{trend}			0.0003	0.026		0.039	0.44
Invasive margin							
Q1	236	95	1 (referent)	1 (referent)	142	1 (referent)	1 (referent)
Q2	225	57	0.56 (0.41–0.78)	0.58 (0.41–0.81)	108	0.70 (0.54–0.90)	0.71 (0.55–0.92)
Q3	225	51	0.52 (0.37–0.74)	0.62 (0.43–0.89)	106	0.71 (0.56–0.92)	0.75 (0.57–0.98)
Q4	226	45	0.45 (0.31–0.64)	0.60 (0.40–0.88)	99	0.63 (0.49–0.81)	0.78 (0.59–1.03)
P_{trend}			<0.0001	0.0059		0.0008	0.091
IRF4^{-} B cells							
Tumor center							
Q1	228	85	1 (referent)	1 (referent)	136	1 (referent)	1 (referent)
Q2	228	52	0.52 (0.37–0.74)	0.79 (0.55–1.14)	104	0.63 (0.49–0.82)	0.81 (0.62–1.06)
Q3	228	60	0.60 (0.43–0.84)	0.76 (0.53–1.07)	109	0.67 (0.52–0.86)	0.81 (0.62–1.05)
Q4	228	51	0.54 (0.38–0.77)	0.66 (0.45–0.96)	106	0.69 (0.54–0.89)	0.81 (0.62–1.07)
P_{trend}			0.0012	0.030		0.0092	0.13
Invasive margin							
Q1	228	79	1 (referent)	1 (referent)	125	1 (referent)	1 (referent)
Q2	228	73	0.92 (0.67–1.26)	1.06 (0.77–1.48)	118	0.93 (0.73–1.20)	0.97 (0.75–1.25)
Q3	228	48	0.58 (0.41–0.83)	0.72 (0.49–1.06)	110	0.83 (0.64–1.07)	0.89 (0.67–1.17)
Q4	228	48	0.57 (0.40–0.82)	0.76 (0.51–1.13)	102	0.76 (0.58–0.98)	0.94 (0.71–1.25)
P_{trend}			0.0002	0.070		0.023	0.56
HLA-DR^{+} B cells							
Tumor center							
Q1	228	79	1 (referent)	1 (referent)	125	1 (referent)	1 (referent)
Q2	228	56	0.67 (0.48–0.95)	0.78 (0.55–1.12)	115	0.88 (0.68–1.13)	0.96 (0.74–1.24)
Q3	228	65	0.76 (0.55–1.05)	0.85 (0.60–1.21)	112	0.83 (0.64–1.07)	0.86 (0.66–1.13)
Q4	228	48	0.56 (0.39–0.80)	0.69 (0.47–1.02)	103	0.77 (0.59–0.99)	0.89 (0.67–1.17)
P_{trend}			0.0044	0.099		0.041	0.31
Invasive margin							
Q1	228	78	1 (referent)	1 (referent)	126	1 (referent)	1 (referent)
Q2	228	76	0.95 (0.69–1.30)	1.01 (0.73–1.39)	122	0.94 (0.73–1.21)	0.91 (0.71–1.18)
Q3	228	46	0.56 (0.39–0.81)	0.79 (0.53–1.17)	103	0.77 (0.59–1.00)	0.89 (0.67–1.19)
Q4	228	48	0.57 (0.40–0.82)	0.69 (0.46–1.03)	104	0.75 (0.58–0.98)	0.87 (0.65–1.15)
P_{trend}			0.0001	0.046		0.012	0.33
HLA-DR^{-} B cells							
Tumor center							
Q1	228	77	1 (referent)	1 (referent)	131	1 (referent)	1 (referent)
Q2	228	61	0.70 (0.50–0.98)	0.62 (0.44–0.88)	106	0.69 (0.53–0.89)	0.69 (0.53–0.90)
Q3	228	58	0.68 (0.48–0.96)	0.61 (0.42–0.88)	115	0.78 (0.61–1.00)	0.75 (0.58–0.98)
Q4	228	52	0.62 (0.44–0.89)	0.54 (0.37–0.79)	103	0.71 (0.54–0.91)	0.68 (0.52–0.89)
P_{trend}			0.0088	0.0017		0.024	0.011
Invasive margin							
Q1	228	81	1 (referent)	1 (referent)	125	1 (referent)	1 (referent)
Q2	228	67	0.82 (0.59–1.13)	0.87 (0.62–1.21)	119	0.95 (0.74–1.22)	0.98 (0.76–1.26)
Q3	228	50	0.60 (0.42–0.85)	0.83 (0.57–1.20)	108	0.82 (0.64–1.07)	0.95 (0.73–1.25)
Q4	228	50	0.60 (0.42–0.85)	0.75 (0.51–1.10)	103	0.79 (0.60–1.02)	0.95 (0.72–1.26)
P_{trend}			0.0009	0.14		0.039	0.71

(continued)

IRF4⁺HLA-DR⁺ B cells							
Tumor center							
Q1	382	126	1 (referent)	1 (referent)	203	1 (referent)	1 (referent)
Q2	177	56	0.91 (0.66–1.25)	0.89 (0.64–1.23)	98	0.99 (0.78–1.26)	0.96 (0.75–1.24)
Q3	176	36	0.58 (0.40–0.85)	0.90 (0.62–1.33)	81	0.80 (0.62–1.04)	1.07 (0.82–1.40)
Q4	177	30	0.47 (0.32–0.70)	0.62 (0.41–0.95)	73	0.71 (0.54–0.93)	0.87 (0.65–1.16)
P_{trend}			<0.0001	0.044		0.0059	0.55
Invasive margin							
Q1	293	113	1 (referent)	1 (referent)	172	1 (referent)	1 (referent)
Q2	206	52	0.60 (0.43–0.83)	0.65 (0.46–0.91)	95	0.70 (0.54–0.90)	0.71 (0.55–0.91)
Q3	206	42	0.49 (0.34–0.70)	0.62 (0.43–0.91)	95	0.70 (0.55–0.90)	0.78 (0.59–1.02)
Q4	207	41	0.46 (0.32–0.66)	0.53 (0.42–0.92)	93	0.67 (0.52–0.86)	0.82 (0.62–1.08)
P_{trend}			<0.0001	0.0060		0.0012	0.15
IRF4⁻HLA-DR⁺ B cells							
Tumor center							
Q1	228	75	1 (referent)	1 (referent)	125	1 (referent)	1 (referent)
Q2	228	60	0.75 (0.53–1.05)	0.81 (0.58–1.15)	116	0.87 (0.67–1.12)	0.94 (0.72–1.21)
Q3	228	61	0.74 (0.53–1.03)	0.79 (0.55–1.12)	106	0.76 (0.59–0.98)	0.81 (0.62–1.06)
Q4	228	52	0.64 (0.45–0.92)	0.72 (0.49–1.05)	108	0.80 (0.62–1.04)	0.88 (0.67–1.16)
P_{trend}			0.017	0.085		0.055	0.23
Invasive margin							
Q1	228	75	1 (referent)	1 (referent)	122	1 (referent)	1 (referent)
Q2	228	76	1.00 (0.73–1.37)	0.98 (0.70–1.36)	126	1.03 (0.80–1.32)	0.96 (0.74–1.24)
Q3	228	49	0.62 (0.43–0.89)	0.81 (0.55–1.19)	103	0.80 (0.61–1.04)	0.87 (0.66–1.16)
Q4	228	48	0.60 (0.42–0.86)	0.69 (0.46–1.02)	104	0.79 (0.61–1.02)	0.90 (0.68–1.20)
P_{trend}			0.0005	0.048		0.022	0.39
IRF4⁻HLA-DR⁻ B cells							
Tumor center							
Q1	228	81	1 (referent)	1 (referent)	136	1 (referent)	1 (referent)
Q2	228	57	0.62 (0.44–0.87)	0.60 (0.42–0.84)	101	0.63 (0.47–0.81)	0.68 (0.52–0.88)
Q3	228	56	0.60 (0.43–0.85)	0.62 (0.43–0.89)	109	0.68 (0.53–0.87)	0.72 (0.56–0.94)
Q4	228	54	0.60 (0.43–0.85)	0.53 (0.36–0.76)	109	0.71 (0.55–0.91)	0.69 (0.53–0.90)
P_{trend}			0.0041	0.0014		0.016	0.014
Invasive margin							
Q1	228	78	1 (referent)	1 (referent)	120	1 (referent)	1 (referent)
Q2	228	66	0.82 (0.59–1.14)	0.88 (0.63–1.23)	119	0.97 (0.75–1.25)	1.01 (0.78–1.31)
Q3	228	50	0.63 (0.44–0.89)	0.86 (0.59–1.25)	112	0.89 (0.69–1.15)	1.03 (0.79–1.36)
Q4	228	54	0.68 (0.48–0.96)	0.88 (0.60–1.29)	104	0.84 (0.65–1.09)	1.04 (0.79–1.39)
P_{trend}			0.0090	0.49		0.15	0.72

CI = Confidence interval, HR = Hazard ratio.

Multivariable Cox proportional hazards regression models were adjusted for sex, age (<65, 65–75, >75), year of operation (2000–2005, 2006–2010, 2011–2015), tumor location (proximal colon, distal colon, rectum), TNM stage (I–II, III, IV), tumor grade (well/moderately differentiated, poorly differentiated), lymphovascular invasion (no, yes), MMR status (proficient, deficient), *BRAF* status (wild-type, V600E mutant), and T cell density score (low, intermediate, high, missing). P_{trend} values were calculated by using the four ordinal categories of B cell densities (Q1–Q4) as continuous variables in univariable and multivariable Cox proportional hazard regression models.

Table S20. Univariable and multivariable Cox regression models for cancer-specific survival and overall survival according to various combinations of plasma cell densities in the tumor center and other immune cell variables in the study cohort.

		Colorectal cancer-specific survival			Overall survival		
	No. of cases	No. of events	Univariable HR (95% CI)	Multivariable HR (95% CI)	No. of events	Univariable HR (95% CI)	Multivariable HR (95% CI)
Plasma cells tumor center – B cells tumor center	912						
Both low	336	106	1 (referent)	1 (referent)	181	1 (referent)	1 (referent)
Either high	240	72	0.86 (0.64–1.16)	0.61 (0.60–1.11)	119	0.82 (0.65–1.04)	0.76 (0.60–0.96)
Both high	336	70	0.61 (0.45–0.82)	0.64 (0.47–0.87)	155	0.77 (0.63–0.96)	0.78 (0.63–0.97)
P_{trend}			0.0012	0.0048		0.020	0.026
Plasma cells tumor center – B cells invasive margin	912						
Both low	265	99	1 (referent)	1 (referent)	153	1 (referent)	1 (referent)
Either high	382	107	0.70 (0.53–0.91)	0.77 (0.58–1.03)	187	0.77 (0.62–0.96)	0.80 (0.64–0.99)
Both high	265	42	0.38 (0.26–0.54)	0.49 (0.34–0.70)	115	0.64 (0.50–0.82)	0.71 (0.55–0.91)
P_{trend}			<0.0001	0.0001		0.0003	0.0058
Plasma cells tumor center – $CD3^+$ T cells tumor center	905						
Both low	308	115	1 (referent)	1 (referent)	184	1 (referent)	1 (referent)
Either high	290	71	0.58 (0.43–0.77)	0.65 (0.48–0.89)	129	0.63 (0.50–0.79)	0.62 (0.50–0.79)
Both high	307	59	0.45 (0.33–0.62)	0.54 (0.39–0.74)	139	0.64 (0.51–0.80)	0.67 (0.53–0.84)
P_{trend}			<0.0001	<0.0001		<0.0001	0.0004
Plasma cells tumor center – $CD3^+$ T cells invasive margin	893						
Both low	273	105	1 (referent)	1 (referent)	159	1 (referent)	1 (referent)
Either high	350	94	0.66 (0.50–0.87)	0.84 (0.63–1.13)	177	0.81 (0.66–1.01)	0.89 (0.71–1.11)
Both high	270	44	0.36 (0.26–0.52)	0.50 (0.35–0.73)	112	0.59 (0.46–0.75)	0.65 (0.51–0.84)
P_{trend}			<0.0001	0.0003		<0.0001	0.0009
Plasma cells tumor center – $CD8^+$ T cells tumor center	903						
Both low	280	103	1 (referent)	1 (referent)	163	1 (referent)	1 (referent)
Either high	343	90	0.65 (0.49–0.87)	0.78 (0.58–1.04)	157	0.70 (0.56–0.88)	0.72 (0.58–0.91)
Both high	280	51	0.44 (0.31–0.61)	0.53 (0.38–0.76)	129	0.67 (0.53–0.85)	0.70 (0.55–0.89)
P_{trend}			<0.0001	0.0004		0.0007	0.0030
Plasma cells tumor center – $CD8^+$ T cells invasive margin	899						
Both low	264	97	1 (referent)	1 (referent)	149	1 (referent)	1 (referent)
Either high	371	101	0.70 (0.53–0.92)	0.75 (0.56–1.00)	182	0.82 (0.66–1.01)	0.85 (0.68–1.06)
Both high	264	44	0.40 (0.28–0.58)	0.53 (0.37–0.76)	115	0.67 (0.53–0.86)	0.72-0.56–0.93)
P_{trend}			<0.0001	0.0005		0.0015	0.010
Plasma cells tumor center – $CD8^-$ T cells tumor center	898						
Both low	298	104	1 (referent)	1 (referent)	298	1 (referent)	1 (referent)
Either high	304	84	0.73 (0.54–0.97)	0.69 (0.52–0.93)	304	0.76 (0.61–0.95)	0.71 (0.57–0.89)
Both high	296	55	0.46 (0.33–0.64)	0.53 (0.38–0.74)	296	0.63 (0.50–0.79)	0.66 (0.52–0.84)
P_{trend}			<0.0001	0.0001		<0.0001	0.0005

(continued)

Plasma cells tumor center – $CD8^-$ T cells invasive margin	888						
Both low	267	98	1 (referent)	1 (referent)	155	1 (referent)	1 (referent)
Either high	357	98	0.71 (0.54–0.94)	0.92 (0.69–1.24)	185	0.84 (0.68–1.04)	0.96 (0.77–1.19)
Both high	264	44	0.39 (0.27–0.55)	0.57 (0.40–0.83)	103	0.55 (0.43–0.71)	0.66 (0.51–0.85)
P_{trend}			<0.0001	0.0046		<0.0001	0.0016
Plasma cells tumor center – Tertiary lymphoid structure density	912						
Both low	268	110	1 (referent)	1 (referent)	164	1 (referent)	1 (referent)
Either high	375	103	0.61 (0.47–0.80)	0.67 (0.51–0.89)	186	0.73 (0.59–0.90)	0.77 (0.62–0.95)
Both high	269	35	0.26 (0.18–0.38)	0.39 (0.26–0.58)	105	0.49 (0.39–0.63)	0.61 (0.47–0.78)
P_{trend}			<0.0001	<0.0001		<0.0001	0.0001

CI = Confidence interval, HR = Hazard ratio.

Multivariable Cox proportional hazards regression models were adjusted for sex, age (<65, 65–75, >75), year of operation (2000–2005, 2006–2010, 2011–2015), tumor location (proximal colon, distal colon, rectum), TNM stage (I–II, III, IV), tumor grade (well/moderately differentiated, poorly differentiated), lymphovascular invasion (no, yes), MMR status (proficient, deficient), and *BRAF* status (wild-type, V600E mutant). P_{trend} values were calculated by using the three ordinal immune cell categories (Both low, Either high, Both high) as continuous variables in univariable and multivariable Cox proportional hazard regression models.

Table S21. Univariable and multivariable Cox regression models for cancer-specific according to various combinations of plasma cell densities in the tumor center and other immune cell variables in the study cohort.

	No. of cases	No. of events	Univariable HR (95% CI)	Multivariable 1 HR (95% CI)	Multivariable 2 HR (95% CI)
**Comparison 1**	912				
Plasma cells tumor center					
Q1	228	85	1 (referent)	1 (referent)	1 (referent)
Q2	228	64	0.68 (0.49–0.94)	0.69 (0.50–0.97)	0.66 (0.47–0.94)
Q3	228	56	0.58 (0.42–0.82)	0.61 (0.41–0.88)	0.64 (0.44–0.93)
Q4	228	43	0.44 (0.31–0.64)	0.43 (0.28–0.68)	0.47 (0.30–0.73)
P_{trend}			<0.0001	0.0003	0.0010
B cells tumor center					
Q1	228	83	1 (referent)	1 (referent)	1 (referent)
Q2	228	52	0.56 (0.40–0.80)	0.64 (0.45–0.91)	0.80 (0.64–1.36)
Q3	228	61	0.65 (0.47–0.90)	0.88 (0.61–1.27)	0.93 (0.64–1.36)
Q4	228	52	0.58 (0.41–0.82)	0.92 (0.61–1.41)	0.94 (0.62–1.42)
P_{trend}			0.0046	0.81	0.83
**Comparison 2**	912				
Plasma cells tumor center					
Q1	228	85	1 (referent)	1 (referent)	1 (referent)
Q2	228	64	0.68 (0.49–0.94)	0.74 (0.53–1.03)	0.64 (0.45–0.90)
Q3	228	56	0.58 (0.42–0.82)	0.63 (0.45–0.89)	0.62 (0.43–0.88)
Q4	228	43	0.44 (0.31–0.64)	0.51 (0.35–0.75)	0.48 (0.32–0.72)
P_{trend}			<0.0001	0.0003	0.0005
B cells invasive margin					
Q1	228	82	1 (referent)	1 (referent)	1 (referent)
Q2	228	74	0.90 (0.66–1.23)	0.98 (0.71–1.34)	1.10 (0.79–1.54)
Q3	228	43	0.50 (0.34–0.72)	0.56 (0.39–0.82)	0.70 (0.47–1.04)
Q4	228	49	0.56 (0.39–0.80)	0.67 (0.46–0.97)	0.89 (0.60–1.31)
P_{trend}			<0.0001	0.0025	0.17
**Comparison 3**	905				
Plasma cells tumor center					
Q1	228	85	1 (referent)	1 (referent)	1 (referent)
Q2	226	63	0.67 (0.49–0.93)	0.74 (0.53–1.03)	0.65 (0.46–0.92)
Q3	227	56	0.59 (0.42–0.82)	0.70 (0.49–1.00)	0.66 (0.46–0.96)
Q4	224	41	0.43 (0.29–0.62)	0.56 (0.37–0.84)	0.49 (0.32–0.75)
P_{trend}			<0.0001	0.0052	0.0020
$CD3^+$ T cells tumor center					
Q1	227	80	1 (referent)	1 (referent)	1 (referent)
Q2	226	73	0.88 (0.64–1.20)	0.97 (0.70–1.34)	1.37 (0.98–1.93)
Q3	226	53	0.59 (0.42–0.84)	0.69 (0.48–1.00)	1.03 (0.71–1.51)
Q4	226	39	0.43 (0.29–0.63)	0.56 (0.36–0.85)	0.76 (0.50–1.17)
P_{trend}			<0.0001	0.0019	0.19
**Comparison 4**	893				
Plasma cells tumor center					
Q1	224	83	1 (referent)	1 (referent)	1 (referent)
Q2	226	63	0.68 (0.49–0.94)	0.77 (0.55–1.07)	0.68 (0.48–0.95)
Q3	224	55	0.59 (0.42–0.83)	0.71 (0.50–1.01)	0.70 (0.49–1.01)
Q4	219	42	0.45 (0.31–0.66)	0.60 (0.41–0.89)	0.57 (0.38–0.85)
P_{trend}			<0.0001	0.0084	0.0081
$CD3^+$ T cells invasive margin					
Q1	224	87	1 (referent)	1 (referent)	1 (referent)
Q2	223	71	0.77 (0.56–1.05)	0.82 (0.60–1.12)	0.95 (0.68–1.31)
Q3	223	50	0.52 (0.37–0.73)	0.57 (0.40–0.81)	0.75 (0.52–1.08)
Q4	223	35	0.34 (0.23–0.50)	0.40 (0.26–0.59)	0.62 (0.40–0.96)
P_{trend}			<0.0001	<0.0001	0.019

(continued)

**Comparison 5**	903				
Plasma cells tumor center					
Q1	226	84	1 (referent)	1 (referent)	1 (referent)
Q2	226	63	0.68 (0.49–0.94)	0.73 (0.52–1.02)	0.64 (0.46–0.91)
Q3	225	55	0.58 (0.41–0.82)	0.65 (0.46–0.93)	0.64 (0.45–0.93)
Q4	226	42	0.43 (0.30–0.63)	0.51 (0.35–0.75)	0.48 (0.32–0.72)
P_{trend}			<0.0001	0.0006	0.0006
CD8^{+} T cells tumor center					
Q1	226	77	1 (referent)	1 (referent)	1 (referent)
Q2	226	72	0.90 (0.65–1.25)	1.00 (0.72–1.38)	1.23 (0.88–1.72)
Q3	226	53	0.64 (0.45–0.91)	0.74 (0.51–1.06)	0.98 (0.68–1.43)
Q4	225	42	0.50 (0.34–0.72)	0.62 (0.42–0.92)	0.82 (0.54–1.24)
P_{trend}			<0.0001	0.0060	0.29
**Comparison 6**	899				
Plasma cells tumor center					
Q1	224	83	1 (referent)	1 (referent)	1 (referent)
Q2	226	62	0.67 (0.48–0.93)	0.74 (0.53–1.03)	0.66 (0.47–0.92)
Q3	226	56	0.60 (0.42–0.84)	0.67 (0.48–0.95)	0.69 (0.49–0.99)
Q4	223	41	0.43 (0.30–0.63)	0.53 (0.36–0.78)	0.50 (0.34–0.74)
P_{trend}			<0.0001	0.0010	0.0009
CD8^{+} T cells invasive margin					
Q1	225	95	1 (referent)	1 (referent)	1 (referent)
Q2	225	55	0.49 (0.35–0.69)	0.52 (0.37–0.72)	0.75 (0.53–1.06)
Q3	225	51	0.46 (0.33–0.65)	0.52 (0.37–0.73)	0.70 (0.49–1.00)
Q4	224	41	0.38 (0.26–0.54)	0.43 (0.30–0.63)	0.65 (0.44–0.96)
P_{trend}			<0.0001	<0.0001	0.021
**Comparison 7**	898				
Plasma cells tumor center					
Q1	226	84	1 (referent)	1 (referent)	1 (referent)
Q2	225	63	0.68 (0.49–0.94)	0.73 (0.53–1.02)	0.68 (0.48–0.95)
Q3	224	55	0.58 (0.42–0.82)	0.67 (0.47–0.95)	0.66 (0.47–0.95)
Q4	223	41	0.43 (0.30–0.62)	0.53 (0.37–0.83)	0.52 (0.34–0.80)
P_{trend}			<0.0001	0.0020	0.0022
CD8^{-} T cells tumor center					
Q1	225	77	1 (referent)	1 (referent)	1 (referent)
Q2	225	69	0.85 (0.61–1.18)	0.86 (0.60–1.20)	0.96 (0.68–1.36)
Q3	224	59	0.69 (0.49–0.97)	0.78 (0.55–1.11)	0.84 (0.58–1.22)
Q4	224	38	0.43 (0.29–0.63)	0.53 (0.35–0.81)	0.67 (0.44–1.03)
P_{trend}			<0.0001	0.0038	0.054
**Comparison 8**	888				
Plasma cells tumor center					
Q1	222	82	1 (referent)	1 (referent)	1 (referent)
Q2	225	62	0.67 (0.48–0.94)	0.74 (0.53–1.03)	0.66 (0.47–0.92)
Q3	223	55	0.60 (0.42–0.84)	0.69 (0.49–0.98)	0.69 (0.49–0.99)
Q4	218	41	0.44 (0.31–0.65)	0.56 (0.38–0.83)	0.57 (0.38–0.85)
P_{trend}			<0.0001	0.0026	0.0050
CD8^{-} T cells invasive margin					
Q1	222	84	1 (referent)	1 (referent)	1 (referent)
Q2	222	66	0.76 (0.55–1.05)	0.75 (0.54–1.04)	0.88 (0.64–1.23)
Q3	222	54	0.58 (0.41–0.82)	0.65 (0.44–0.88)	1.03 (0.72–1.48)
Q4	222	36	0.36 (0.24–0.53)	0.41 (0.28–0.62)	0.60 (0.39–0.91)
P_{trend}			<0.0001	<0.0001	0.063

(continued)

**Comparison 9**	912				
Plasma cells tumor center					
Q1	228	85	1 (referent)	1 (referent)	1 (referent)
Q2	228	64	0.68 (0.49–0.94)	0.79 (0.57–1.10)	0.76 (0.54–1.06)
Q3	228	56	0.58 (0.42–0.82)	0.74 (0.52–1.04)	0.69 (0.49–0.98)
Q4	228	43	0.44 (0.31–0.64)	0.60 (0.41–0.87)	0.56 (0.38–0.83)
P_{trend}			<0.0001	0.0067	0.0032
Tertiary lymphoid structure density					
Q1	228	97	1 (referent)	1 (referent)	1 (referent)
Q2	228	78	0.74 (0.55–1.00)	0.78 (0.58–1.06)	0.73 (0.53–0.99)
Q3	228	39	0.33 (0.23–0.48)	0.37 (0.25–0.53)	0.48 (0.32–0.71)
Q4	228	34	0.28 (0.19–0.42)	0.32 (0.21–0.47)	0.51 (0.33–0.77)
P_{trend}			<0.0001	<0.0001	<0.0001

CI = Confidence interval, HR = Hazard ratio.

Multivariable Cox proportional hazards regression models were adjusted for sex, age (<65, 65–75, >75), year of operation (2000–2005, 2006–2010, 2011–2015), tumor location (proximal colon, distal colon, rectum), TNM stage (I–II, III, IV), tumor grade (well/moderately differentiated, poorly differentiated), lymphovascular invasion (no, yes), MMR status (proficient, deficient), and *BRAF* status (wild-type, V600E mutant). P_{trend} values were calculated by using the four ordinal categories of immune cell variables (Q1–Q4) as continuous variables in univariable and multivariable Cox proportional hazard regression models.

Table S22. Clinicopathological characteristics of colorectal cancer cases according to the overall plasma cell and B cell densities in the tumor center and invasive margin in the validation cohort.

		Overall plasma cell density (cells/mm^2) Median (25th–75th percentiles)				Overall B cell density (cells/mm^2) Median (25th–75th percentiles)			
Characteristic	Total *n*	CT	*P*	IM	*P*	CT	*P*	IM	*P*
All cases	737	35 (9.3-126)		53 (13-160)		6.2 (1.8-22)		21 (4.9-75)	
Sex			0.18		0.08		0.18		0.38
Female	347	32 (9.7–105)		61 (14–176)		5.2 (1.4–22)		21 (5.6–81)	
Male	390	39 (9.2–138)		47 (13–147)		6.8 (1.9–21)		22 (4.8–71)	
Age (years)			0.15		0.45		0.39		0.45
<65	222	29 (8.4–90)		52 (12–156)		6.4 (1.6–24)		26 (6.0–77)	
65–75	271	44 (11–127)		47 (12–177)		6.5 (2.0–23)		18 (4.2–70)	
>75	244	36 (9.4–244)		61 (16–165)		5.2 (1.8–19)		22 (5.4–73)	
Year of operation			0.94		0.40		0.79		0.050
2006–2010	140	39 (13–108)		46 (7.6–158)		5.6 (2.2–19)		13 (3.6–49)	
2011–2015	206	32 (7.6–136)		52 (15–199)		6.7 (1.8–26)		20 (5.1–88)	
2016–2020	391	37 (8.7–126)		55 (16–150)		6.4 (1.7–21)		24 (5.5–71)	
Tumor location			0.015		0.013		0.23		0.00078
Proximal colon	312	46 (11–141)		71 (18–189)		5.7 (1.7–22)		24 (6.3–86)	
Distal colon	197	33 (5.8–119)		47 (11–138)		5.5 (1.4–18)		14 (3.2–40)	
Rectum	228	28 (9.3–89)		47 (7.9–151)		7.8 (2.1–25)		26 (5.4–84)	
AJCC stage			<0.0001		<0.0001		<0.0001		<0.0001
I	170	58 (21–179)		85 (21–215)		13 (3.1–43)		40 (15–119)	
II	247	42 (9.5–137)		53 (14–173)		5.5 (1.8–17)		19 (4.7–60)	
III	241	26 (7.9–98)		60 (16–159)		5.5 (1.5–20)		19 (5.0–66)	
IV	79	13 (2.9–59)		13 (3.7–41)		3.0 (1.1–9.8)		5.3 (1.2–30)	
Tumor grade			0.49		0.28		0.92		0.16
Low-grade	630	36 (9.3–126)		53 (14–165)		6.1 (1.8–21)		20 (4.8–69)	
High-grade	107	32 (7.1–107)		46 (9.6–130)		6.5 (1.1–31)		28 (6.6–107)	
Lymphovascular invasion			0.0008		<0.0001		0.042		0.0085
No	401	45 (11–153)		72 (20–210)		7.0 (2.1–24)		25 (6.6–79)	
Yes	336	28 (7.0–88)		38 (7.8–110)		5.2 (1.3–18)		14 (3.8–69)	
MMR status			0.098		0.0003		0.16		<0.0001
MMR proficient	618	33 (8.7–122)		49 (11–150)		6.1 (1.8–20)		18 (4.1–56)	
MMR deficient	119	47 (12–183)		84 (23–293)		8.1 (1.5–36)		46 (14–151)	
BRAF status			0.29		0.24		0.91		0.050
Wild-type	635	34 (8.9–125)		52 (12–159)		6.2 (1.8–21)		20 (4.5–66)	
V600E mutant	102	40 (10–136)		60 (19–187)		5.3 (1.4–23)		30 (7.7–110)	

AJCC = American Joint Committee on Cancer, IM = Invasive margin, MMR = Mismatch repair, CT = Tumor center.
P-values were calculated using either Wilcoxon test (two groups) or Kruskal-Wallis test (three/four groups).

Table S23. Univariable and multivariable Cox regression models for cancer-specific survival and overall survival according to CD138+ plasma cell densities in the tumor center and invasive margin in the validation cohort.

		Colorectal cancer-specific survival			Overall survival		
	No. of cases	No. of events	Univariable HR (95% CI)	Multivariable HR (95% CI)	No. of events	Univariable HR (95% CI)	Multivariable HR (95% CI)
Tumor center							
Q1	183	46	1 (referent)	1 (referent)	65	1 (referent)	1 (referent)
Q2	183	35	0.75 (0.48–1.16)	0.75 (0.48–1.18)	59	0.88 (0.62–1.26)	0.88 (0.61–1.26)
Q3	183	27	0.55 (0.34–0.88)	0.53 (0.32–0.89)	63	0.88 (0.62–1.25)	0.82 (0.57–1.18)
Q4	183	31	0.63 (0.40–0.99)	0.63 (0.39–1.02)	64	0.87 (0.62–1.23)	0.89 (0.62–1.27)
P_{trend}			0.018	0.031		0.46	0.47
Invasive margin							
Q1	178	47	1 (referent)	1 (referent)	70	1 (referent)	1 (referent)
Q2	178	34	0.74 (0.48–1.15)	0.93 (0.59–1.49)	56	0.81 (0.57–1.16)	0.89 (0.62–1.28)
Q3	178	24	0.50 (0.31–0.82)	0.75 (0.44–1.29)	50	0.70 (0.49–1.01)	0.87 (0.60–1.27)
Q4	177	31	0.64 (0.41–1.00)	0.87 (0.53–1.42)	68	0.92 (0.66–1.28)	1.02 (0.72–1.44)
P_{trend}			0.017	0.44		0.48	0.93

CI = Confidence interval, HR = Hazard ratio.

Multivariable Cox proportional hazards regression models were adjusted for sex, age (<65, 65–75, >75), year of operation (2000–2005, 2006–2010, 2011–2015), tumor location (proximal colon, distal colon, rectum), TNM stage (I–II, III, IV), tumor grade (well/moderately differentiated, poorly differentiated), lymphovascular invasion (no, yes), MMR status (proficient, deficient), *BRAF* status (wild-type, V600E mutant), and T cell density score (low, intermediate, high). P_{trend} values were calculated by using the four ordinal categories of plasma cell densities (Q1–Q4) as continuous variables in univariable and multivariable Cox proportional hazard regression models.

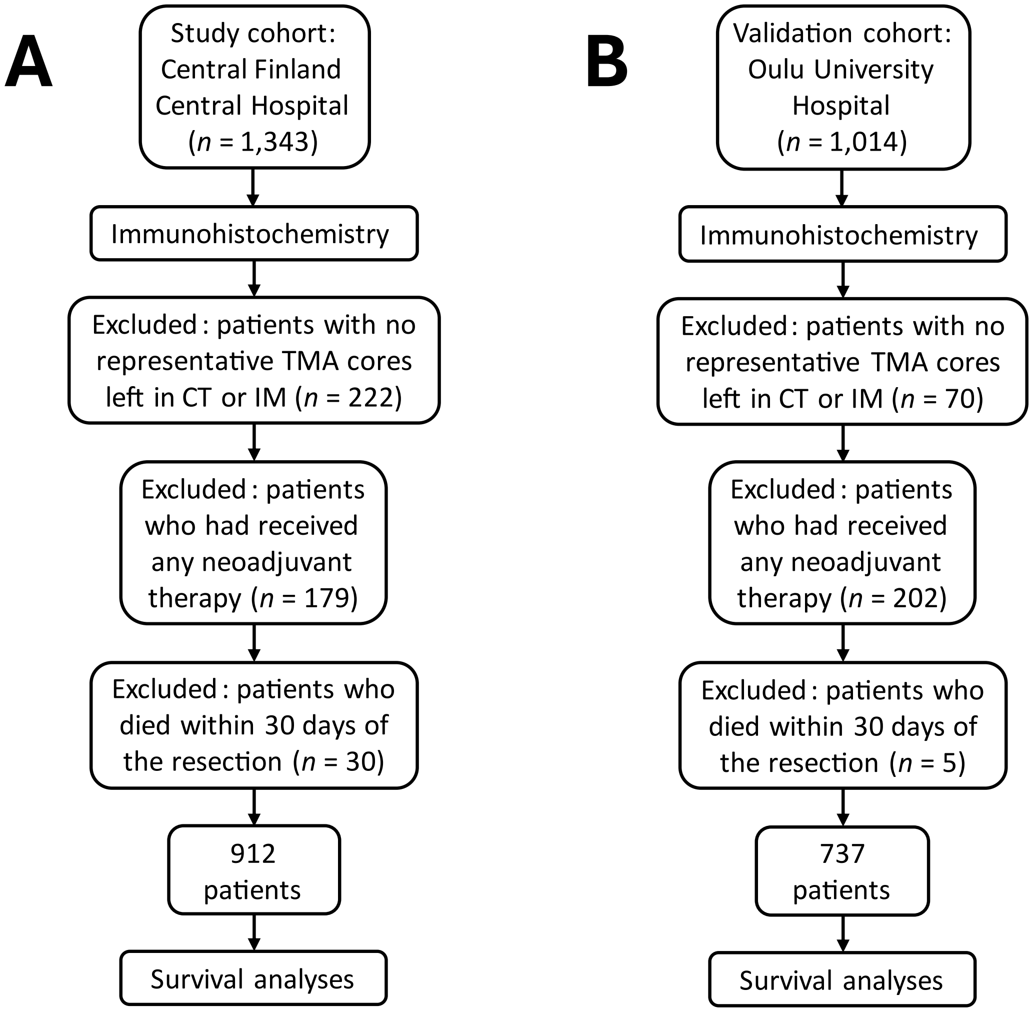

Figure S1. Flow-charts of patient inclusion for the main study cohort (A) and the validation cohort (B).

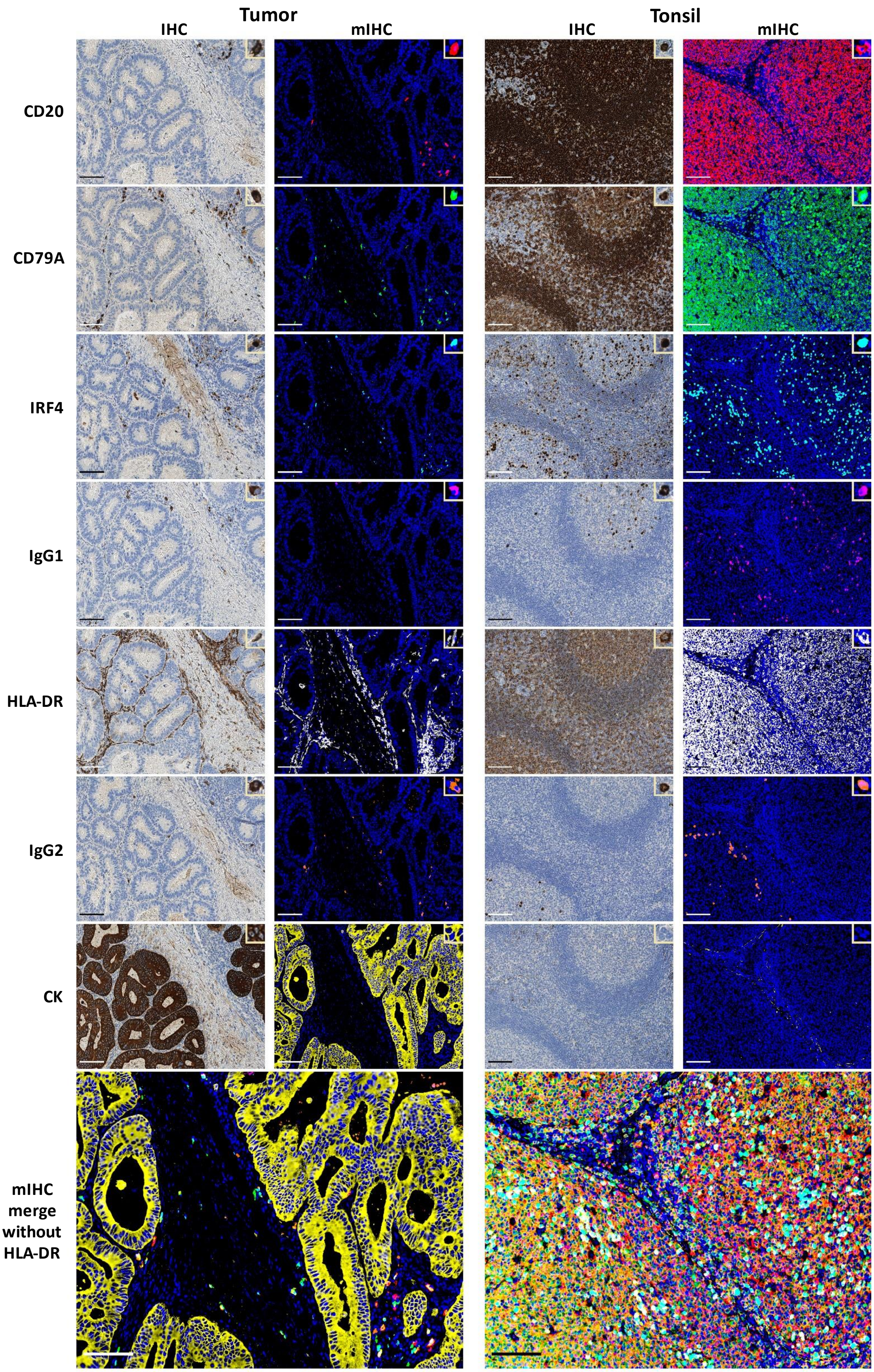

Figure S2. Comparison of immunostaining patterns between standard IHC and multiplex IHC. The scale bar is 100 µm.

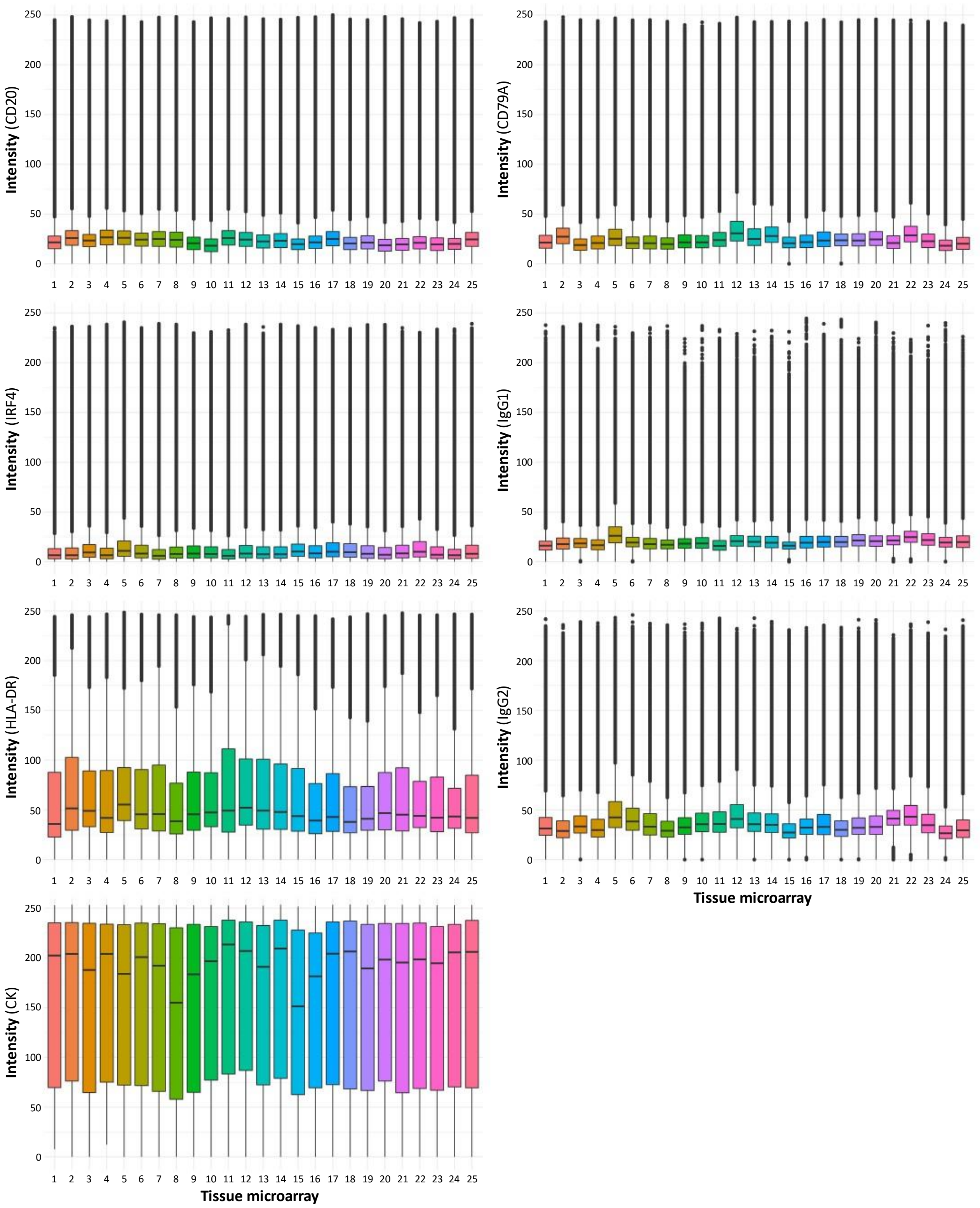

Figure S3. Immunostaining marker intensities across all 25 tissue microarray sections belonging to the main study cohort.

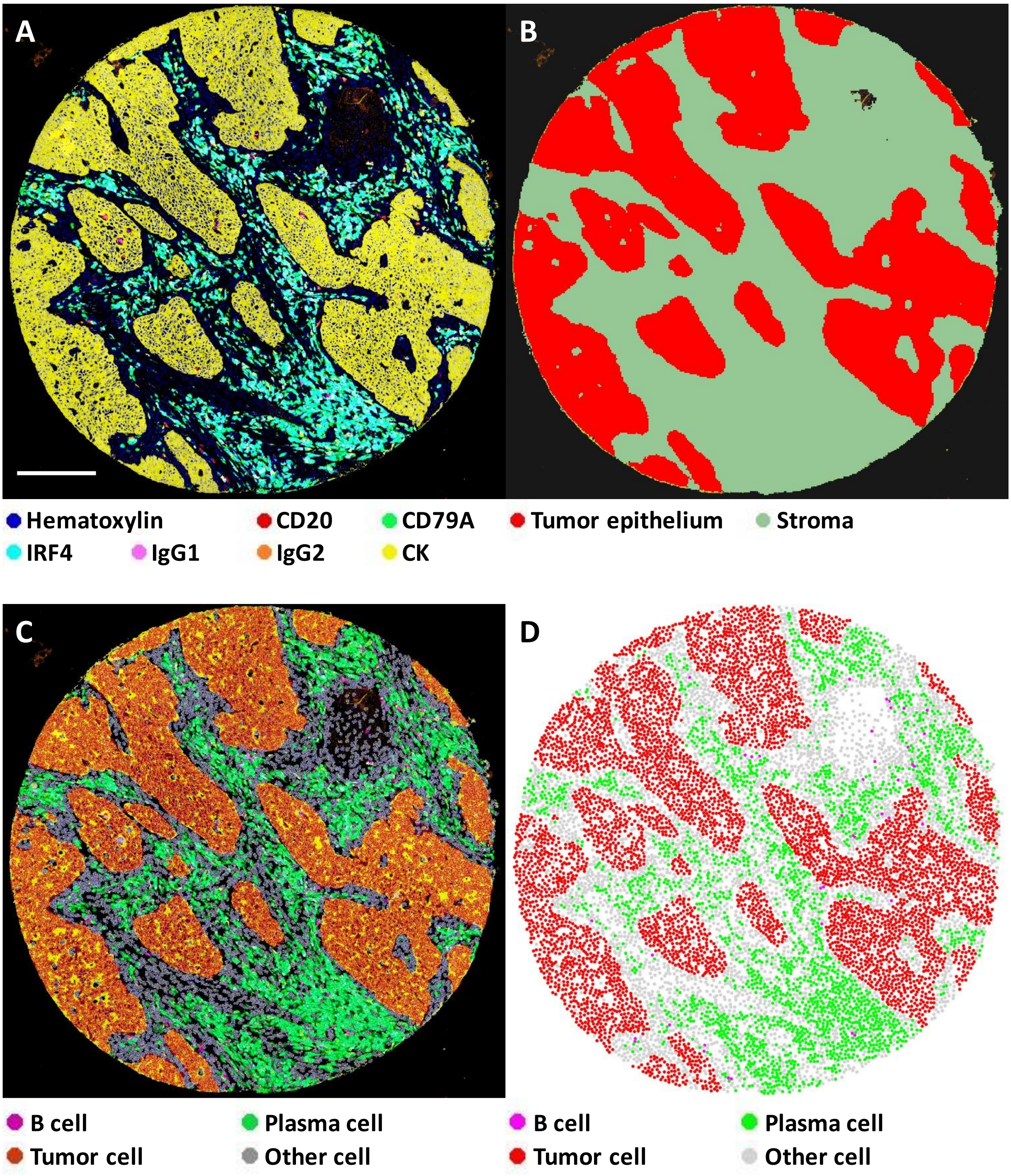

Figure S4. Visual representation of tissue categorization and cell detection output in an example core of the study cohort. A: Multiplex immunohistochemistry image, B: Tissue category classification, C: Cell detection and classification, and D: Cell map of the cell types detected. The scale bar is 200 μm.

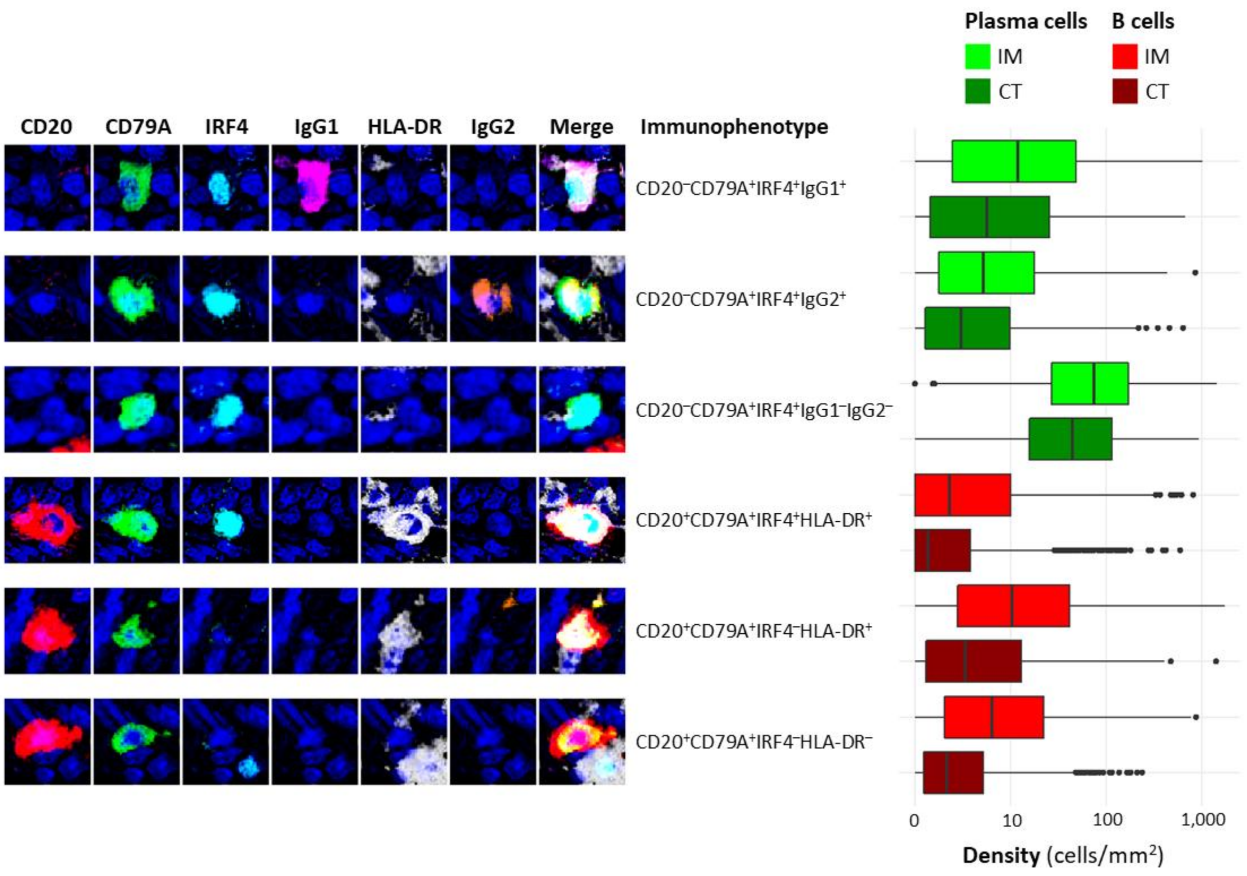

Figure S5. Representative examples of multiplex immunohistochemistry images of the studied plasma cell and B cell subsets together with their densities in invasive margin (IM) and tumor center (CT).

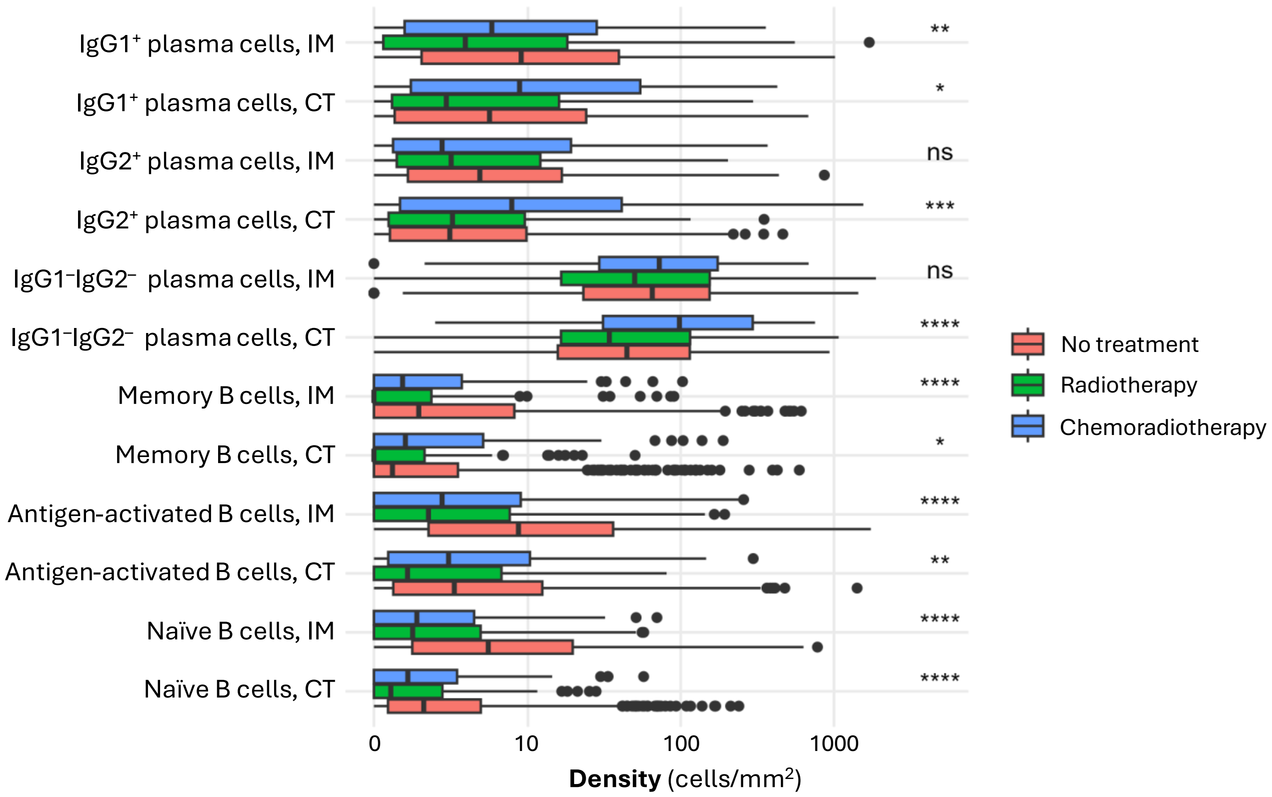

Figure S6. Densities of various plasma cell and B cell subsets in the invasive margin (IM) and tumor center (CT) of MMR proficient patients (n = 971) belonging to the main study cohort: no neoadjuvant treatment (n = 795), short radiotherapy (n = 95) and long chemoradiotherapy (n = 81). Patients with MMR deficient tumors (n = 150) were excluded from the analysis due to the low number of treated individuals with MMR deficient tumors (n = 2). P-values: **** <0.0001, *** <0.001, ** <0.01, * <0.05, and ns >0.05.

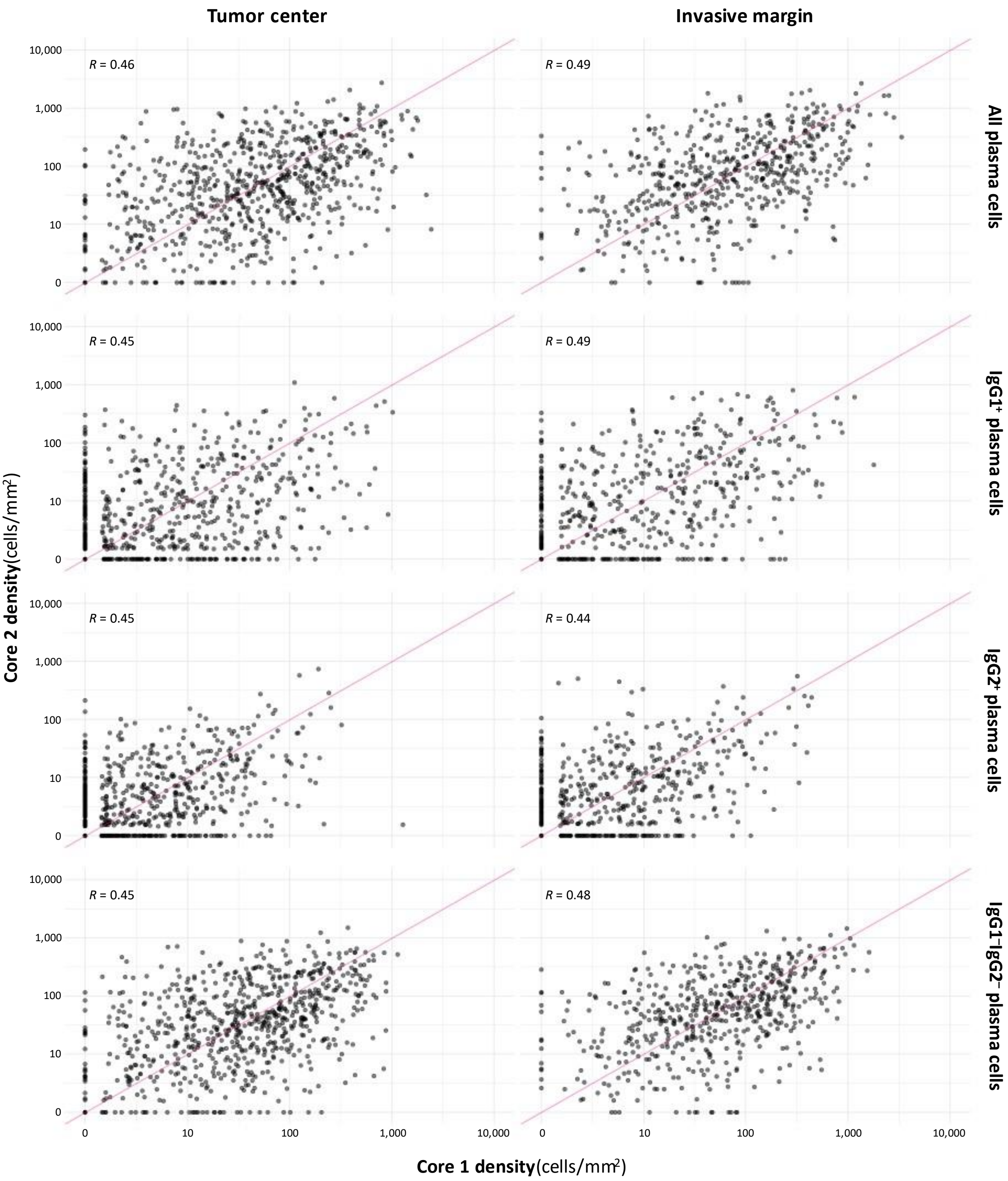

Figure S7. Core-to-core correlations of various plasma cell subsets with the corresponding Spearman's correlation coeffients (*R*) in two randomly chosen cores of tumors with two or more cores.

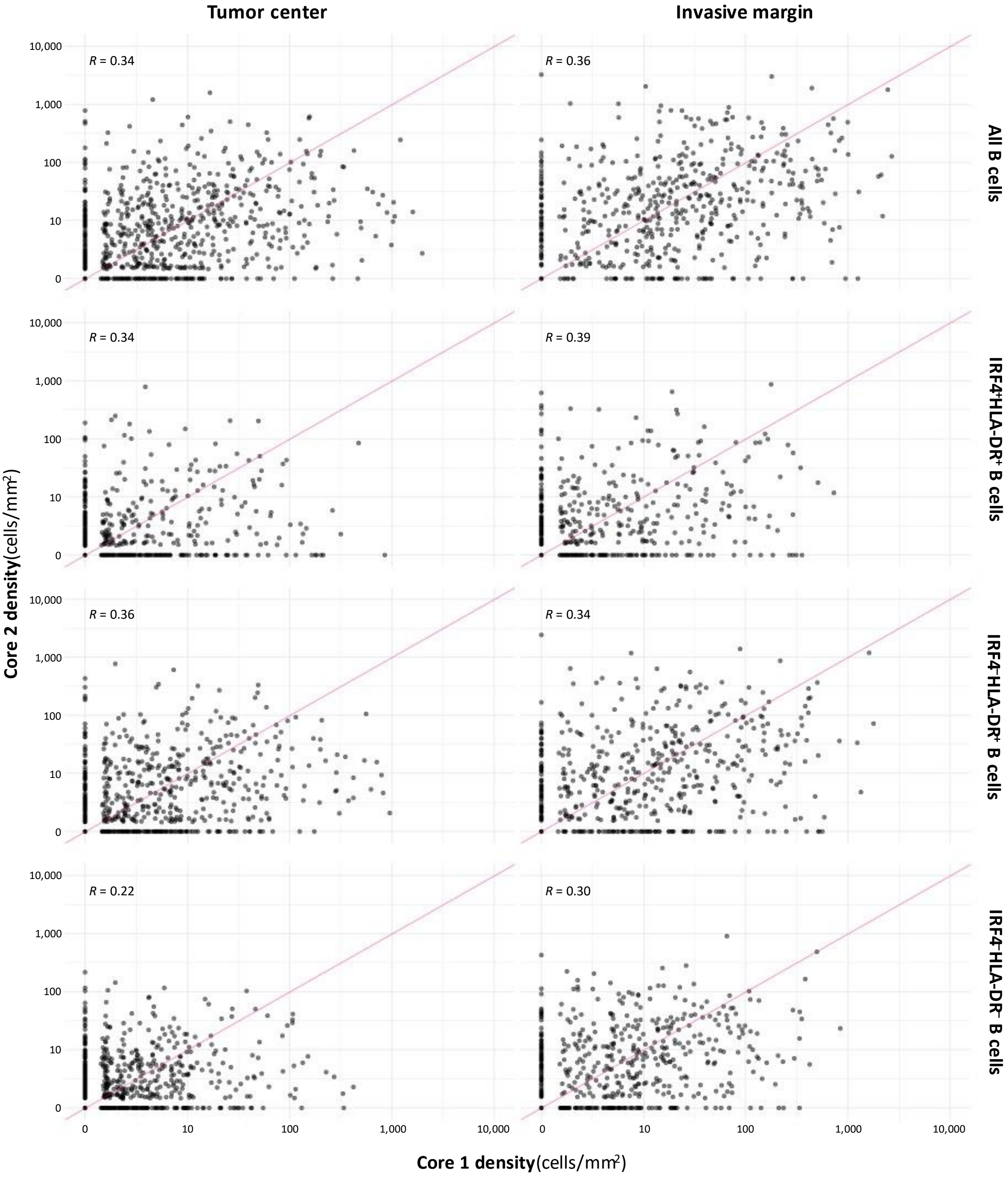

Figure S8. Core-to-core correlations of various B cell subsets with the corresponding Spearman's correlation coefficients (*R*) in two randomly chosen cores of tumors with two or more cores.

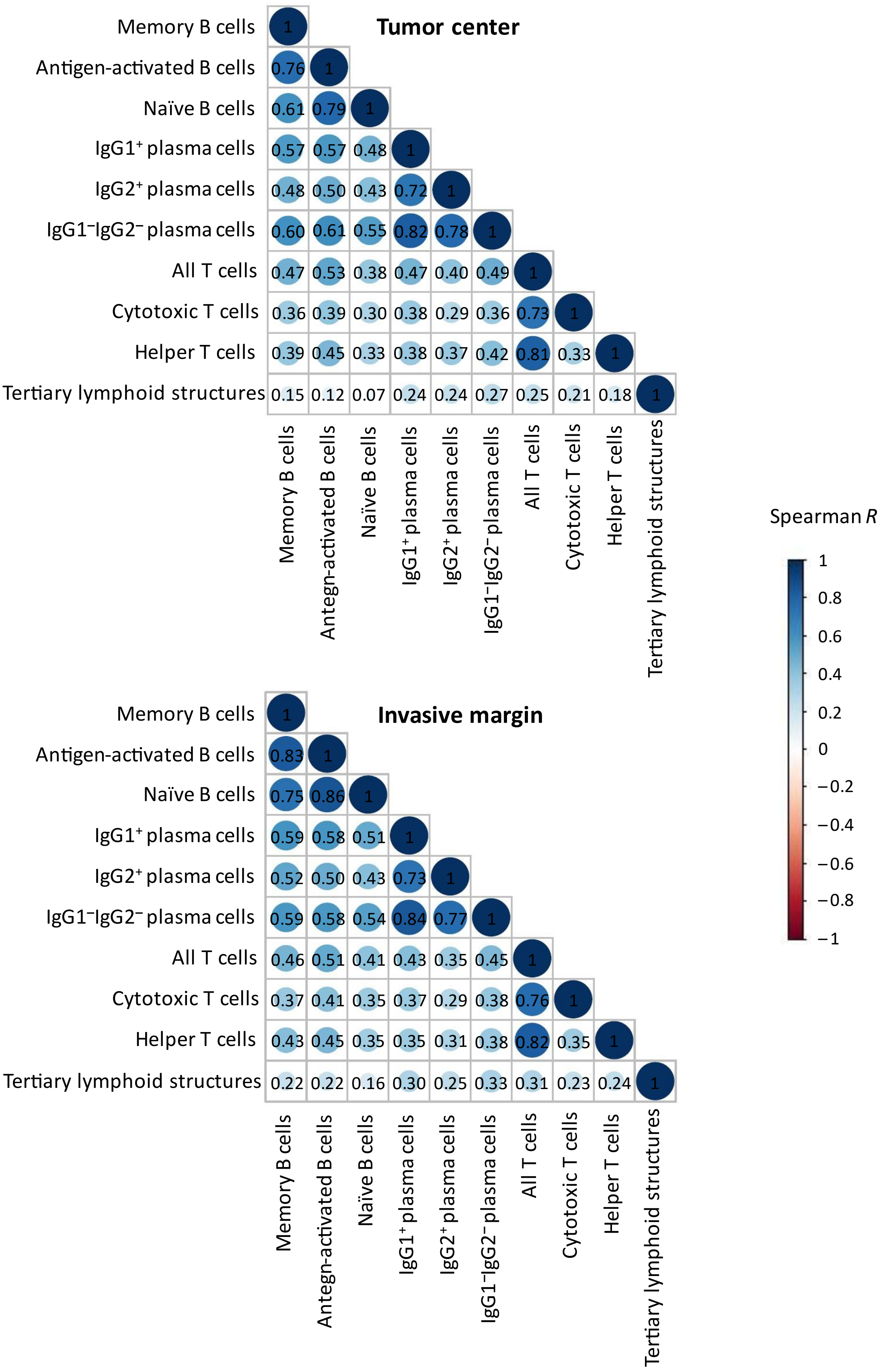

Figure S9. Correlograms depicting correlations between different plasma and B cell subset densities and their relation to the other analyzed immune cell parameters in the center of tumor and invasive margin.

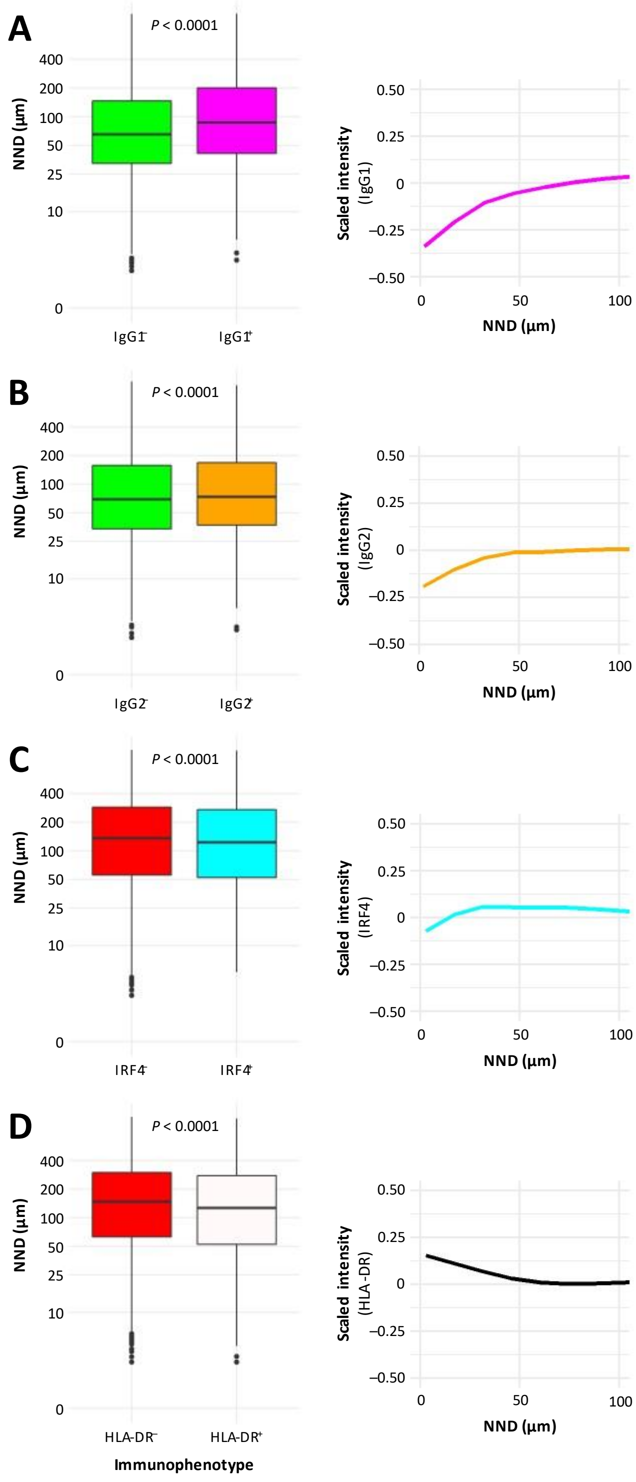

Figure S10. Spatial analyses of plasma cell B and cell subpopulations. The boxplots present nearest neighbor distances (NND) from each plasma cell (n = 501,460) (A–B) or B cell (n = 184,653) (C–D) to the closest tumor cell across all tumors (n = 912). The line plots show scaled intensities of IgG1, IgG2, IRF4, and HLA-DR according to NNDs from immune cell to the closest tumor cell. The plots were generated using generalized additive model smoothing (formula=y ~ s(x)).

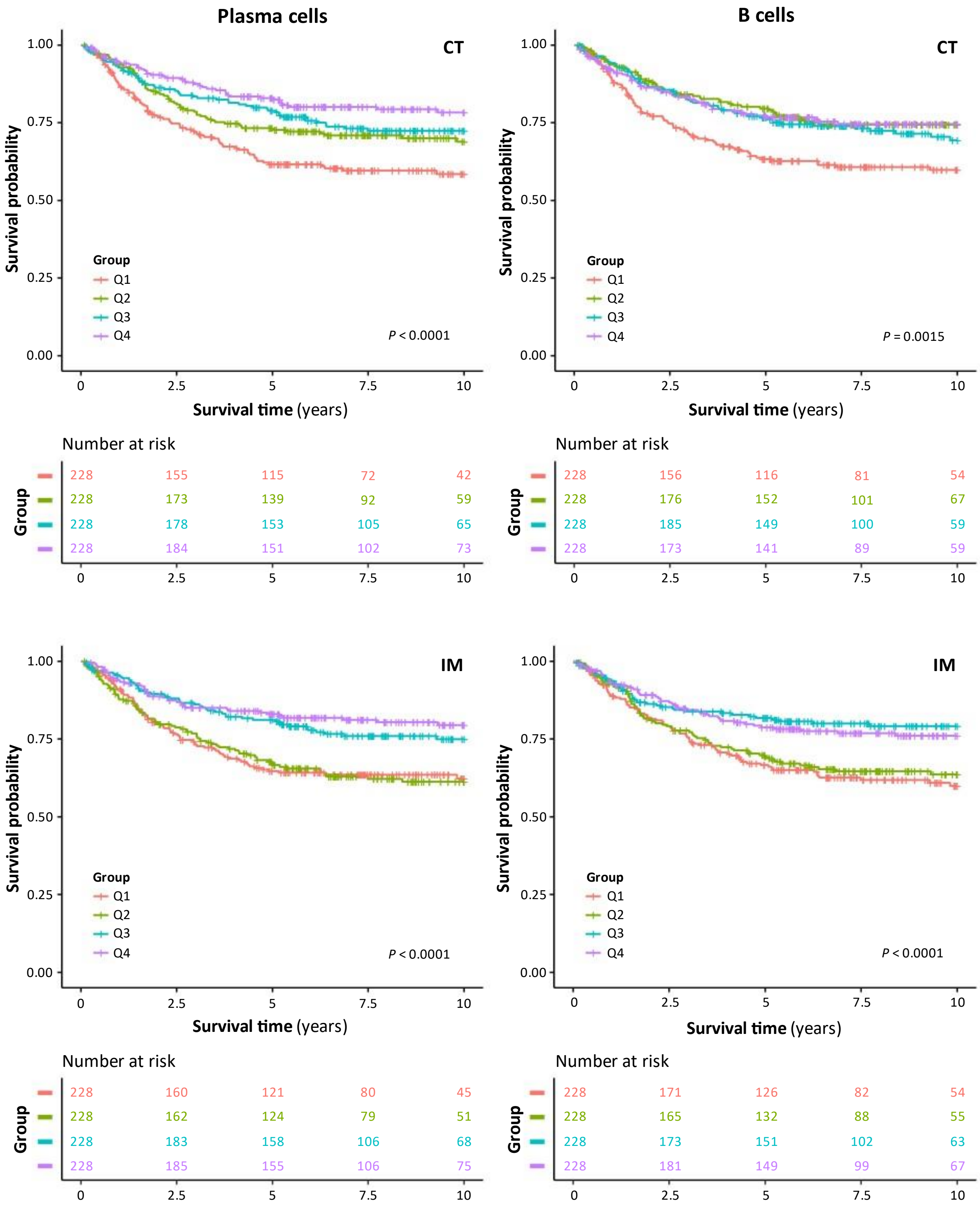

Figure S11. Kaplan-Meier curves depicting the cancer-specific survival according to ordinal quartile categories (Q1–Q4) of the stromal plasma cell and B cell densities in the tumor center (CT) and invasive margin (IM). *P*-values were calculated using the log-rank test.

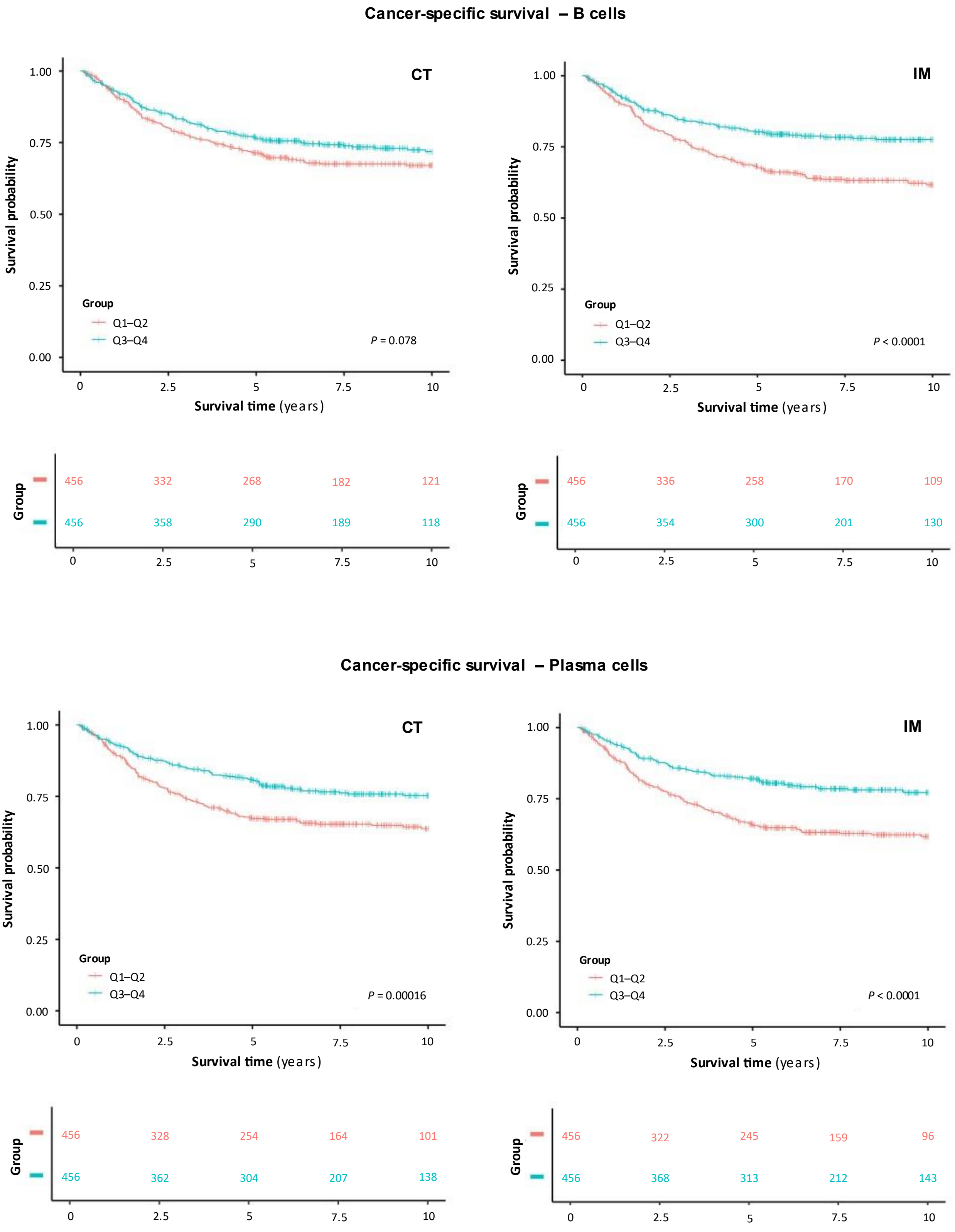

Figure S12. Kaplan-Meier curves depicting the cancer-specific survival for the below (Q1–Q2) and above (Q3–Q4) median categories of overall plasma cell and B cell densities in the tumor center (CT) and invasive margin (IM). *P*-values were calculated using the log-rank test.

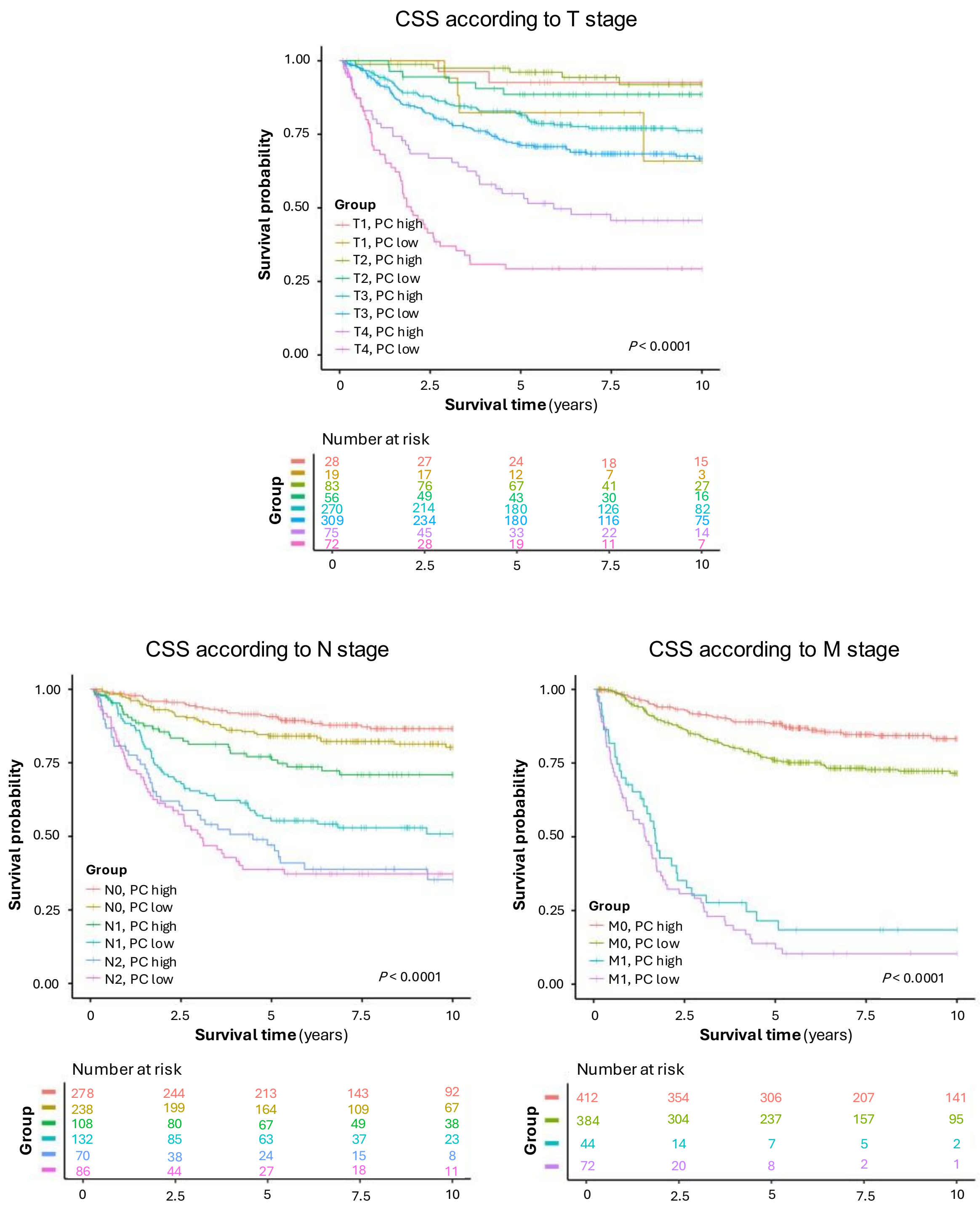

Figure S13. Kaplan-Meier curves depicting cancer-specific survival (CSS) for above (PC high) and below (PC low) median categories of overall plasma cell densities in the tumor center according to T (T1–T4), N (N0–N2), and M (M0–M1) stages. *P*-values were calculated using the log-rank test.

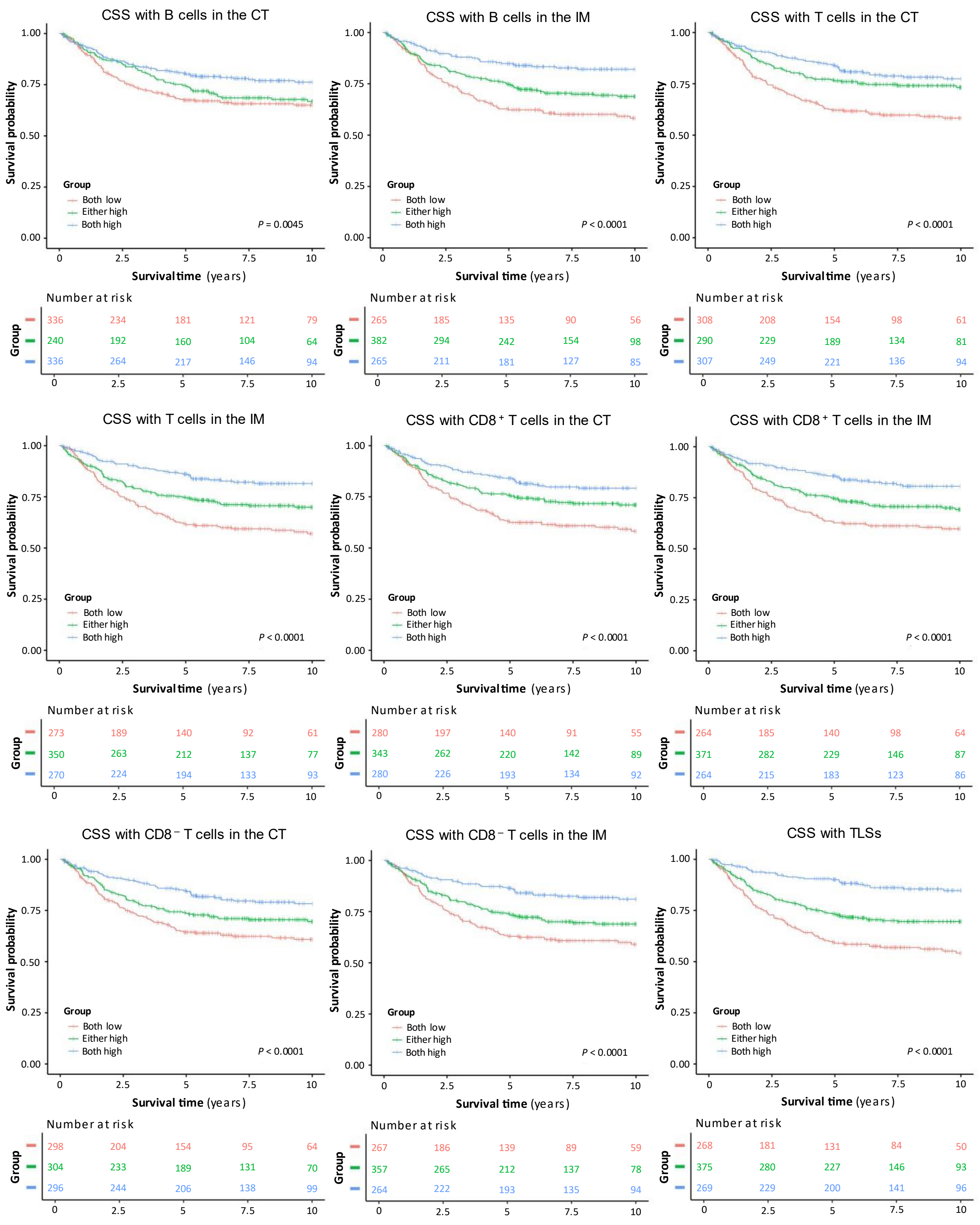

Figure S14. Kaplan-Meier curves depicting cancer-specific survival (CSS) for the above (high) and below (low) median categories of overall plasma cell densities in the tumor center combined with the densities of B cells, all T cells, cytotoxic ($CD8^+$) T cells, helper ($CD8^-$) T cells, or tertiary lymphoid structures (TLSs) in similar (high/low) categorization. *P*-values were calculated using the log-rank test.

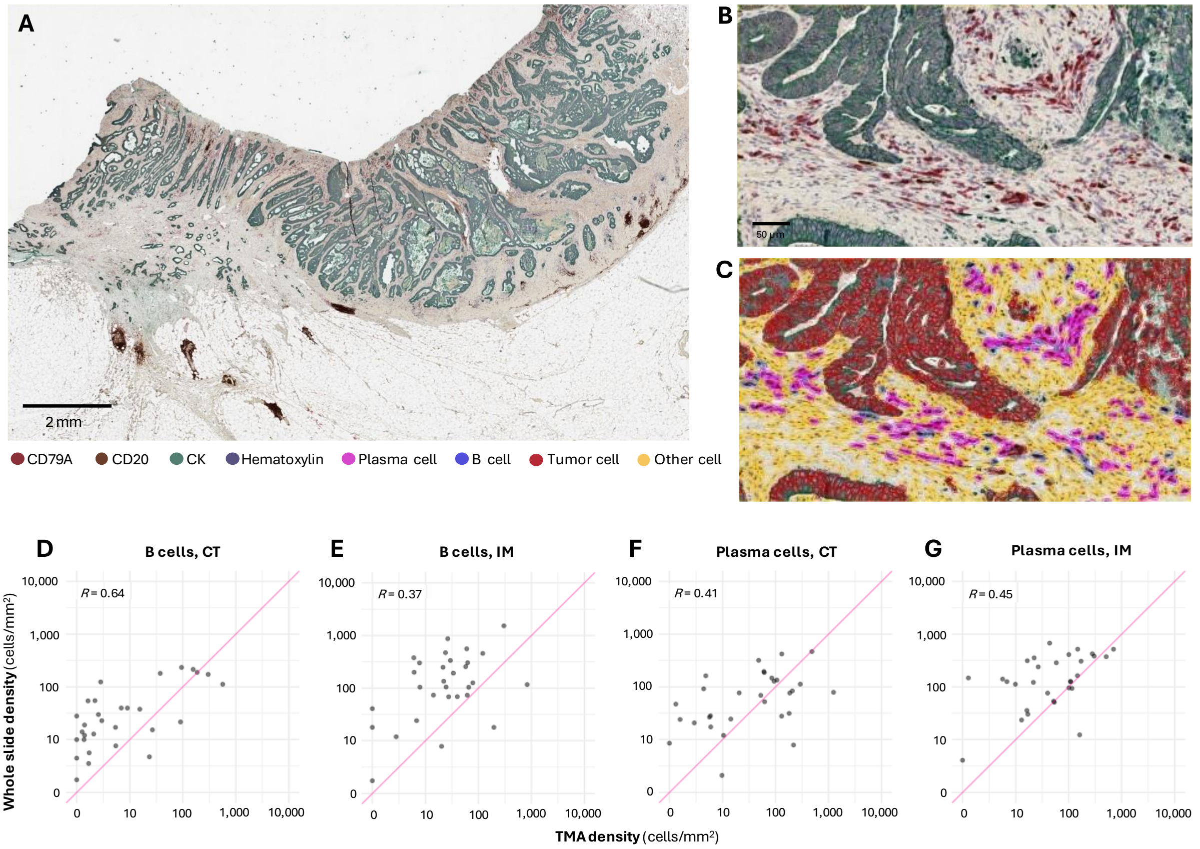

Figure S15. Example of a whole slide CD20-CD79A-CK three-plex immunohistochemistry image (A) and a closer-up view (B). The respective image analysis result image shows the cell types identified (C). Correlations (D–G) between B cell and plasma cell densities calculated in tissue microarray (TMA) cores and whole slides in the tumor center (CT) and invasive margin (IM). Whole slide *n* = 30.

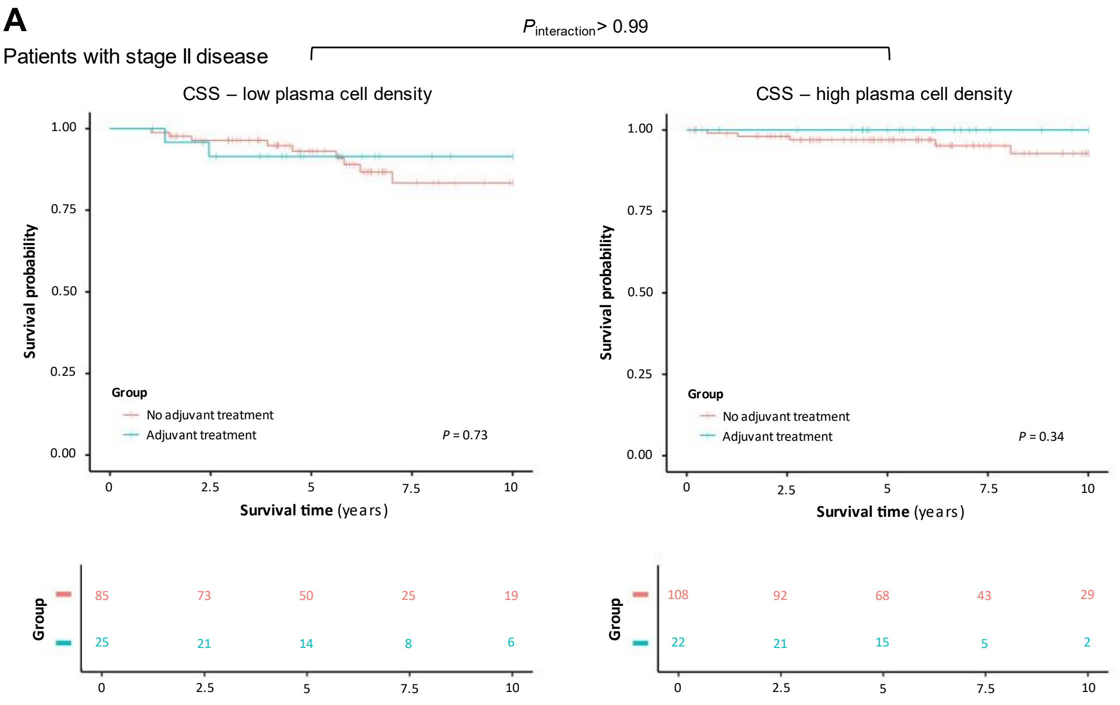

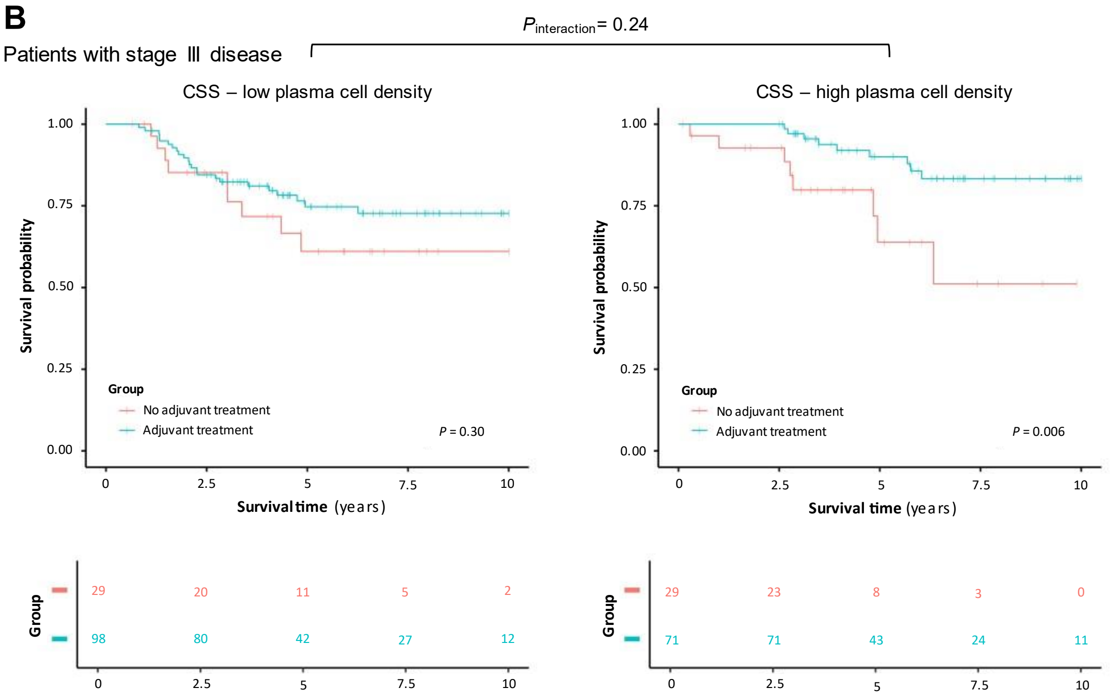

Figure S16. Association between adjuvant treatment status and survival across disease stages (II/III) and plasma cell densities in the tumor center. The analysis included 240 stage II and 227 stage III patients from the validation cohort with available adjuvant treatment data. Among them, 216 received adjuvant treatment (47 in stage II and 169 in stage III), with 120 receiving oxaliplatin-based therapy (e.g., XELOX or FOLFOX), 81 receiving fluoropyrimidine alone, and 15 receiving other treatments (e.g., chemoradiotherapy). Plasma cell densities in the tumor center were dichotomized using the median as cut-off. $P_{interaction}$ values were calculated using the Wald test for the cross product of plasma cell density (low vs. high) and adjuvant treatment status (no vs. yes) in Cox regression models. CSS = cancer-specific survival

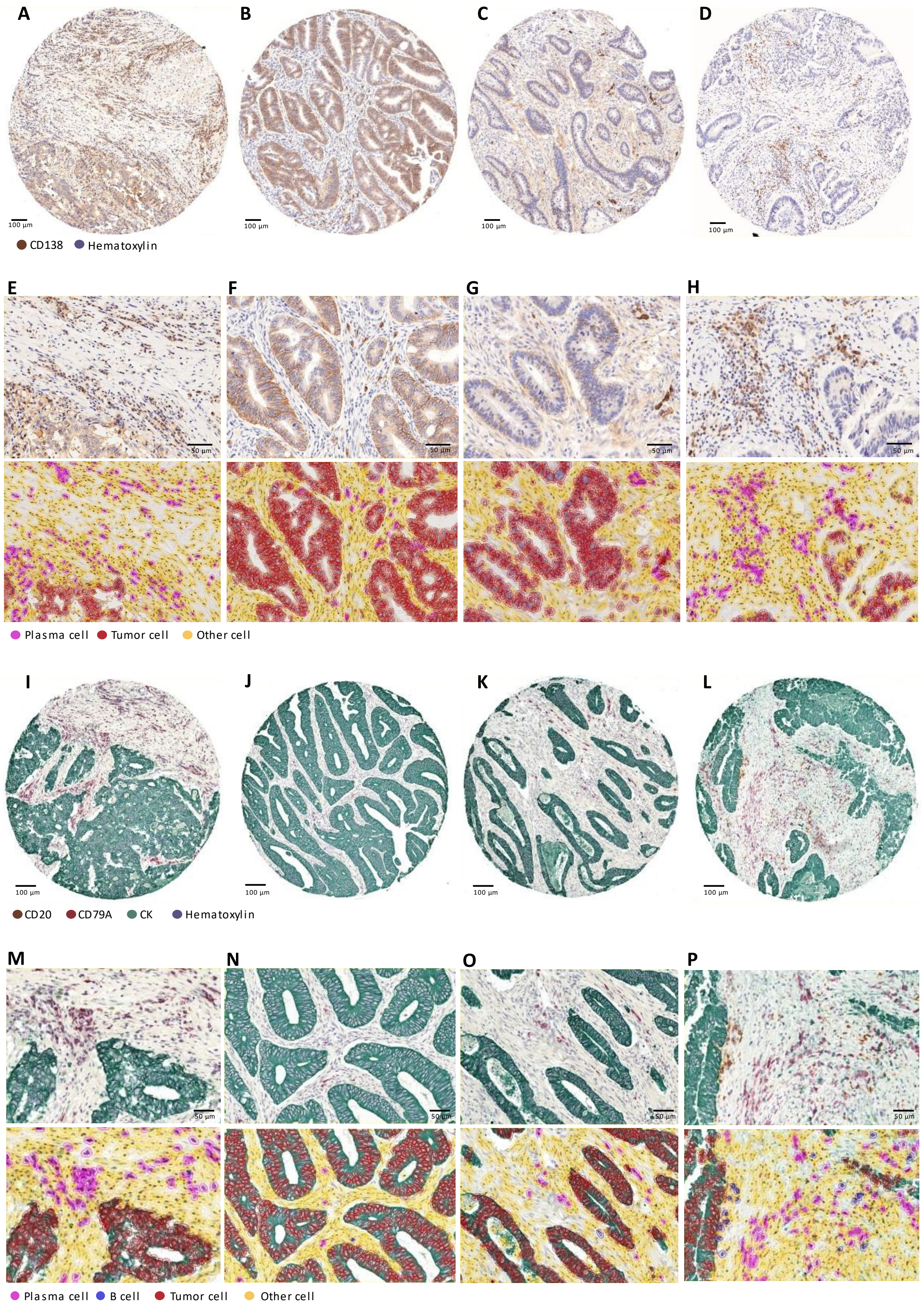

Figure S17. Comparison of CD138 immunohistochemistry and CD20-CD79A-CK three-plex immunohistochemistry for plasma cell identification. Examples of CD138 single-color immunohistochemistry in four TMA cores (A–D) and closer-magnification images along with the image analysis result images (E–H). Examples of CD20-CD79A-CK three-plex immunohistochemistry (I–J) on the same TMA cores as A–D, and closer-magnification images of both the immunohistochemistry along with the image analysis result images (M–P).

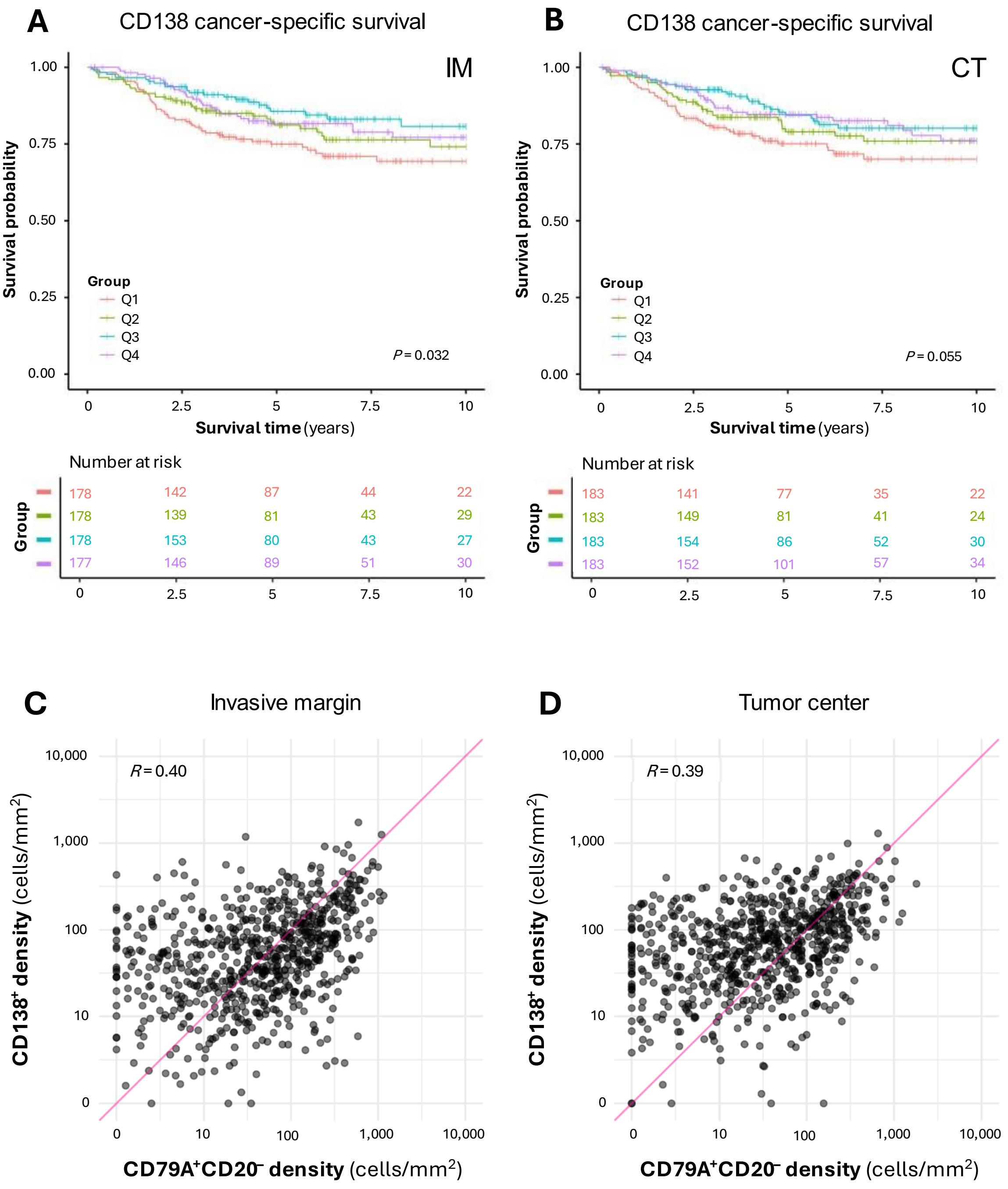

Figure S18. Kaplan-Meier curves depicting colorectal cancer-specific survival according to plasma cell densities detected in the invasive margin (IM) and tumor center (CT) by CD138 immunohistochemistry (A–B). Correlations between plasma cell densities calculated from standard CD138 immunohistochemistry and CD20-CD79A-CK three-plex immunohistochemistry (C–D). CD138: n = 711 in the IM and 732 in the CT.
